# The IgCAM CLMP regulates expression of Connexin43 and Connexin45 in intestinal and ureteral smooth muscle contraction in mice

**DOI:** 10.1242/dmm.032128

**Published:** 2018-02-01

**Authors:** Hanna Langhorst, René Jüttner, Dieter Groneberg, Azadeh Mohtashamdolatshahi, Laura Pelz, Bettina Purfürst, Kai M. Schmidt-Ott, Andreas Friebe, Fritz G. Rathjen

**Affiliations:** 1 Max-Delbrück-Center for Molecular Medicine, DE-13092 Berlin, Germany; 2 Physiologisches Institut der Universität Würzburg, Röntgenring 9, DE-97070 Würzburg, Germany; 3 Charité–Universitätsmedizin Berlin, Department of Nephrology, Charitéplatz 1, DE-10117 Berlin, Germany

**Keywords:** Cell adhesion, IgCAM, CLMP, Congenital short-bowel syndrome, Peristalsis, Hydronephrosis, Connexin43, Connexin45, Smooth muscle cells

## Abstract

CAR-like membrane protein (CLMP), an immunoglobulin cell adhesion molecule (IgCAM), has been implicated in congenital short-bowel syndrome in humans, a condition with high mortality for which there is currently no cure. We therefore studied the function of CLMP in a *Clmp*-deficient mouse model. Although we found that the levels of mRNAs encoding Connexin43 or Connexin45 were not or were only marginally affected, respectively, by *Clmp* deficiency, the absence of CLMP caused a severe reduction of both proteins in smooth muscle cells of the intestine and of Connexin43 in the ureter. Analysis of calcium signaling revealed a disordered cell-cell communication between smooth muscle cells, which in turn induced an impaired and uncoordinated motility of the intestine and the ureter. Consequently, insufficient transport of chyme and urine caused a fatal delay to thrive, a high rate of mortality, and provoked a severe hydronephrosis in CLMP knockouts. Neurotransmission and the capability of smooth muscle cells to contract in ring preparations of the intestine were not altered. Physical obstructions were not detectable and an overall normal histology in the intestine as well as in the ureter was observed, except for a slight hypertrophy of smooth muscle layers. Deletion of *Clmp* did not lead to a reduced length of the intestine as shown for the human *CLMP* gene but resulted in gut malrotations. In sum, the absence of CLMP caused functional obstructions in the intestinal tract and ureter by impaired peristaltic contractions most likely due to a lack of gap-junctional communication between smooth muscle cells.

## Abstract (precis)

**Summary:** The function of the immunoglobulin cell adhesion molecule CLMP was investigated in a mouse model. CLMP is essential for intestinal and ureteral peristalsis, and for expression of Connexin43 and 45 in smooth muscle cells.

## INTRODUCTION

CLMP [CAR-like membrane protein; also termed ACAM (adipocyte adhesion molecule)] is a cell adhesion protein of the immunoglobulin (Ig) superfamily. It has a widespread tissue and organ distribution in mice, with highest expression in the brain and heart based on northern blot data (Raschperger et al., 2004; Eguchi et al., 2005). CLMP colocalizes with the tight-junction markers zonula occludens protein 1 (ZO-1) and occludin in transfected cells (Raschperger et al., 2004; Eguchi et al., 2005; Murakami et al., 2016; Van Der Werf et al., 2012). CLMP induces homotypic aggregation when overexpressed in non-polarized Chinese hamster ovary (CHO) cells (Raschperger et al., 2004), but its *in vivo* function is not well defined. In humans, homozygous and compound heterozygous loss-of-function mutations have been characterized in the *CLMP* gene that correlated with congenital short-bowel syndrome (CSBS), a rare gastrointestinal disorder for which no cure is available (OMIM 615237). These patients have a very short small intestine with a length of approximately 50 cm at birth compared to 190-280 cm in healthy humans (Van Der Werf et al., 2012; Alves et al., 2016; Hamilton et al., 1969; Gonnaud et al., 2016). Of the few patients currently characterized, the best studied mutations – missense mutations in the extracellular domain of CLMP (V124D and C137Y) – resulted in decreased plasma membrane localization of CLMP in transfected cells. Van Der Werf et al. suggested that these mutations might represent null mutations and that CLMP plays a critical role in intestinal development and causes CSBS in humans (Van Der Werf et al., 2012, 2013). Short-bowel syndrome (SBS) in general, as well as CSBS, is combined with severe malnutrition due to a decreased absorptive capacity, malrotation of the intestine, severe delay to thrive and, in several cases, with pyloric hypertrophy. Only 22% of patients survived more than 1 year. Functional obstruction, including disturbed peristalsis, has been discussed as the main source for a shortened intestine (Van Der Werf et al., 2015; Schalamon et al., 1999).

In this study, we have analyzed a *Clmp*-deficient mouse model generated by homologous recombination. Unexpectedly, these mice do not reveal a shortened intestine, but a high rate of *Clmp* mutants died at neonatal and early postnatal stages. This high degree of mortality is likely caused by insufficient transport of nutrients due to impaired motility of the intestinal tract. CLMP deficiency also provoked malrotation of the short bowel and, in addition, a severe bilateral hydronephrosis due to an uncoordinated contraction of the ureter. Although the level of mRNAs encoding Connexin43 or 45 is not or only slightly reduced in the intestine, absence of CLMP strongly decreased expression of both connexins at the protein level in intestinal smooth muscle cells and of Connexin43 in ureteral smooth muscle cells. This reduction resulted in impaired cell-cell communication as shown by the analysis of calcium transients in the smooth muscle layer. Our research revealed a role for CLMP in coordinated transport processes of smooth muscle cells of the intestinal and the urogenital tract. Therefore, these data may provide novel insights into the development of obstructive diseases, which are caused in many cases by contractile dysfunction of smooth muscle cells (Owens et al., 2004).

## RESULTS

### CLMP is expressed in smooth muscle cells of the intestine

**Fig. 1.**
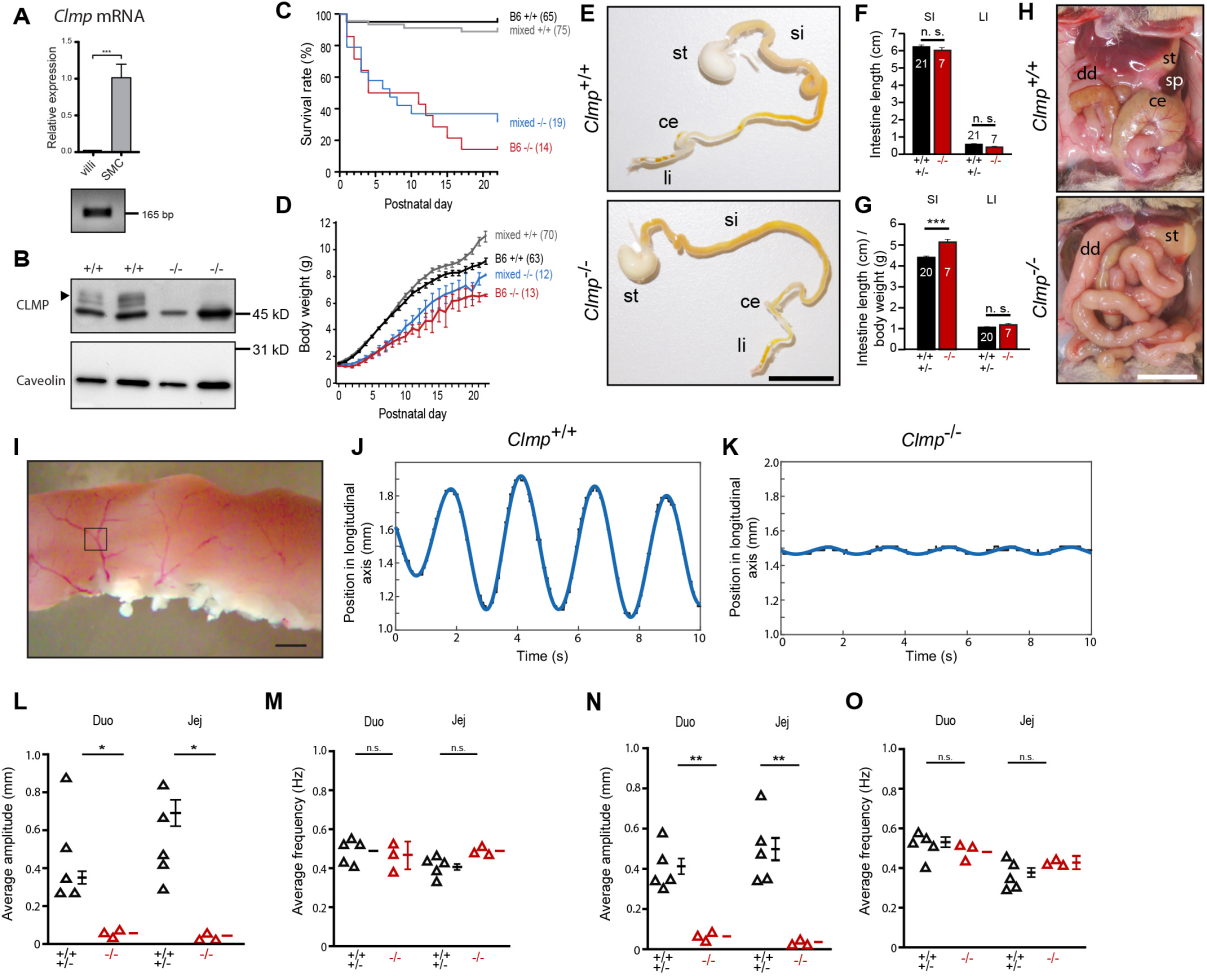
**High mortality and impaired body growth of *Clmp*-deficient mice are caused by intestinal peristalsis deficits.** (A) A *Clmp* transcript is expressed in the intestinal smooth muscle cell layer (SMC) but not in the villi. qRT-PCR from villi and smooth muscle cells and its amplification product are shown. (Data are shown as mean±s.d., *t*-test, *P*=0.0007, *n*=3 per genotype.) (B) Bands at 47 and 48 kDa (arrowhead) are specifically detected by western blotting by an antibody to the cytoplasmic segment of murine CLMP in crude membrane fractions of wild type but not in the knockout smooth muscle cell layer (4 weeks old). An unspecific band at 44 kDa is observed in both genotypes. (C) CLMP-deficient animals (blue, mixed genetic background; red, B6 genetic background) exhibit early postnatal lethality in comparison to control littermates (pooled data of wild-type and heterozygous mice; gray, mixed genetic background; black, B6 genetic background). Numbers in brackets indicate the number of inspected animals. No statistical significance was observed when the survival rates of knockouts between B6 and mixed genetic background were compared (log-rank test, Breslow test or Tarone–Ware test). (D) *Clmp* knockout pups have a delayed body growth. See panel C for color information. Body weight values in grams are shown as means±s.e.m. (E) Dissected gastrointestinal tracts from newborn (P0.5) mice. Note that stomachs of both genotypes are filled with milk, and only the wild-type gut but not the mutant gut contains milk as detected by the white color (see also Fig. 2F). si, small intestine; li, large intestine; st, stomach; ce, cecum. Scale bar: 1 cm. (F,G) Length of the small (SI) and large intestine (LI) of P0.5 CLMP-deficient pups (B6 genetic background) were comparable to wild- type littermates but the length of the small intestine is increased when body weight is taken into account. Length of guts from adult mice are given in Fig. S6. ***P≤0.001. (H) Loss of CLMP results in a mispositioning of the intestine in the mature (P40) mouse abdomen. st, stomach; dd, duodenum; ce, cecum; sp, spleen. Scale bar: 1 cm. (Five wild types and four knockouts at P40-P45, and two wild types and three knockouts at P26-28 were inspected.) (I) Microscopic image of a duodenal segment in an organ bath. The square indicates a branching point of a blood vessel on the intestine, which was used to track intestinal displacements. Scale bar: 1 mm. (J,K) Examples of the amplitude of longitudinal movements of wild-type and CLMP-deficient duodenal segments are shown. Please view Movies 1 and 2 available as supplemental information. (L,M) Amplitude and frequency of longitudinal movements of the duodenum (Duo) and jejunum (Jej) in an organ bath are shown. *P≤0.05. (N,O) Amplitude and frequency of movements perpendicular to the longitudinal direction of the duodenum and jejunum in an organ bath are shown. **P≤0.01.

The phenotype of humans with loss-of-function mutations in the *CLMP* gene indicated a function of the IgCAM CLMP in the intestinal tract (Van Der Werf et al., 2012). CLMP has previously been described as an epithelial cell adhesion protein (Raschperger et al., 2004). However, in our hands, several antibodies to CLMP were not suitable to study the localization of CLMP by immunohistology in the intestine (Fig. S1A). Therefore, initially we performed quantitative real-time PCR (qRT-PCR) of villi and the smooth muscle layer of mouse intestine. *Clmp* mRNA revealed a 4172-fold higher expression in the smooth muscle layer in comparison to villi, suggesting that CLMP might exert its function in the smooth muscle layer of the intestine and not in epithelial cells of the villi (Fig. 1A; Fig. S2 shows the enrichment of these tissue preparations by using cell-type-specific markers in western blotting). *In situ* hybridizations from databases (www.genepaint.org), which revealed *Clmp* mRNA uniformly expressed in the outer layers of the intestine, supported our qRT-PCR findings. Western blotting revealed two bands, at 47 and 48 kDa, in the smooth muscle layer that were not detected in knockout tissue using affinity-purified antibodies to the cytoplasmic segment of murine CLMP (Fig. 1B). In cross-sections of embryonic intestine and ureter, CLMP was predominantly localized in the developing smooth muscle layer as revealed by affinity-purified antibodies to the extracellular domain of CLMP (Fig. S3).

**Fig. 2.**
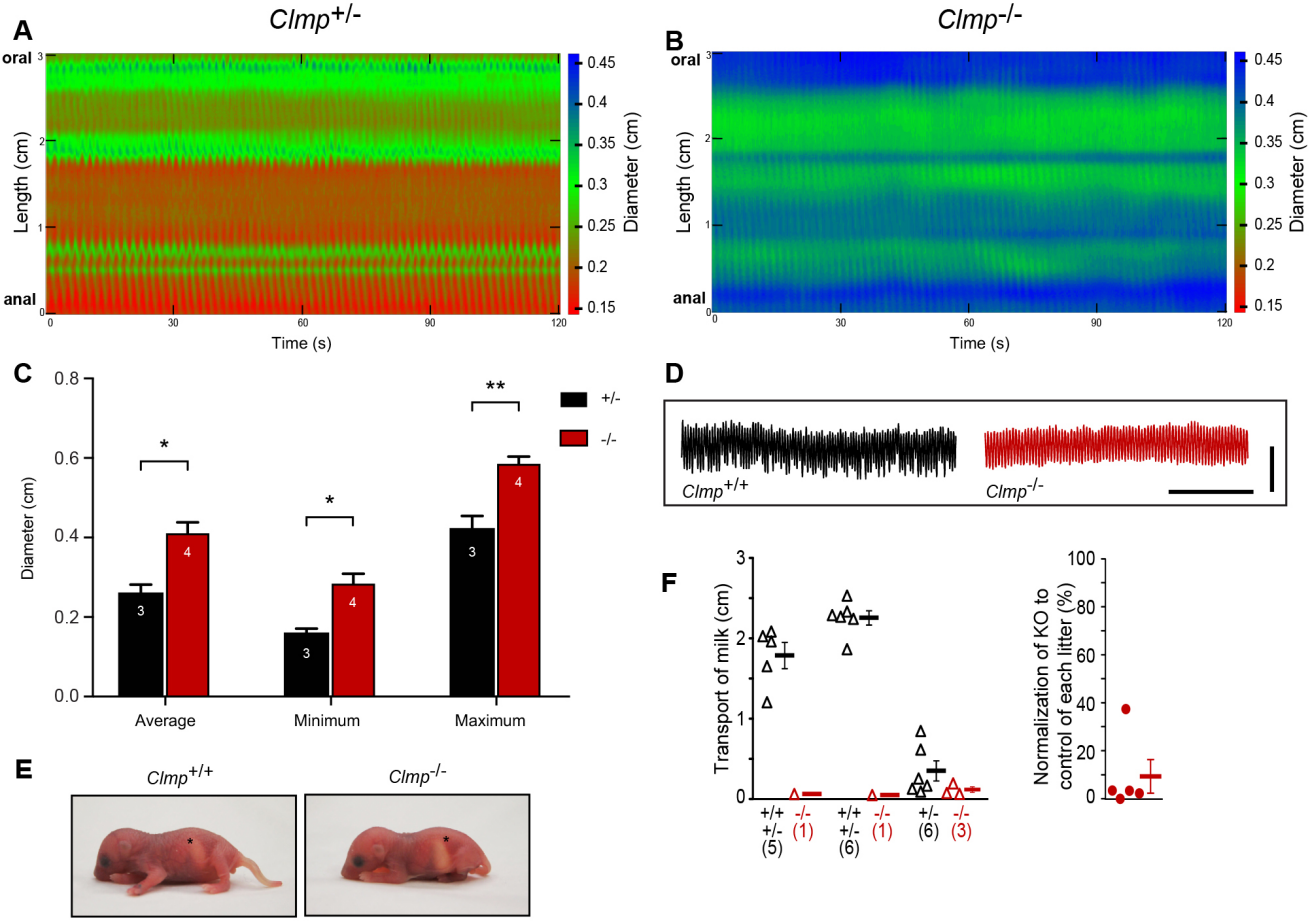
**A severely dilated small intestine resulting in impaired transport of chyme in the absence of CLMP.** (A,B) Spatiotemporal maps of contractile activity in heterozygote and CLMP-deficient duodenum without intraluminal pressure are shown. The diameter of the intestine is expressed in cm in pseudo-color as indicated on the right of each panel (*n*=4 knockouts; *n*=5 heterozygotes or wild type inspected; P42-P48). (C) Summary of duodenum diameter in the organ bath. Numbers in the columns indicate the number of specimens analyzed. *P≤0.05, **P≤0.01. (D) Spontaneous contractility of ring preparations of the duodenum from P2 wild-type (left trace) and *Clmp* mutant (right trace) mice in an organ bath set at resting tension of 1 mN. Similar results were obtained with tissue from P4 mice. Horizontal scale bar: 1 min; vertical scale bar: 0.5 mN. (E) CLMP-deficient neonates at P1.5 display a stomach filled with milk comparable to wild-type controls. Stomachs are marked by asterisks. (Numbers of animals inspected: wild type: 6, heterozygote: 15, knockout: 7). (F) Distance of milk transport from the pylorus along the gut in P0.5 knockout (KO) and control littermates. Values of three litters are presented. (Litter 3 most likely represents pups that had just started to take up milk from the mother, as deduced from the wild types. In wild types, milk was not transported or was transported only a short distance, whereas, in mutants, no transport was detected, as in knockouts from litters 1 and 2). Numbers in brackets indicate the number of analyzed animals. In the right graph, values were normalized to control animals. Controls were set as 100%. See also Fig. 1E on milk transport in the intestine.

### Unlike in humans, CLMP deficiency in mice does not cause shortened intestine, but leads to malrotation, lower body weight and decreased survival

To study the function of the cell adhesion protein CLMP, we generated a global *Clmp* knockout mouse by deleting the start codon containing exon one of the *Clmp* gene. The correct integration of the target vector into the mouse genome was confirmed by Southern blotting and PCR (Fig. S4). Absence of *Clmp* mRNA was demonstrated in brain and intestine by reverse-transcription PCR (RT-PCR) and qRT-PCR, respectively (Fig. S4D,F). Western blotting revealed the absence of CLMP protein in the brain (Fig. S4E), intestine (Fig. 1B) and ureter (see below). The mutant mice were bred on a mixed SV129/C57BL/6 and on a C57BL/6 background. From P1 onwards the body weight of surviving *Clmp*^−/−^ mutants increased much more slowly than that of their control littermates (Fig. 1D and Fig. S5A, for examples). In addition, the survival rate of *Clmp* knockouts drastically decreased after birth (Fig. 1C and Table S1). By trend, this decrease appeared more severe for CLMP-deficient mice on the C57BL/6 background than on the mixed background; however, this did not reach statistical significance. Heterozygous *Clmp* mutants of both strains did not differ in their survival rate or body weight from wild types (Fig. S5B,C; Table S1). The CLMP-deficient mice that reached post-weaning stages gained body weight continuously but reached body weights of littermate controls only at mature stages (Fig. S5D,E).

Loss-of-function mutations in the human *CLMP* gene correlated with a shortened intestine, which causes malabsorption of nutrients, resulting in early lethality (Van Der Werf et al., 2012, 2015). In CLMP-deficient mice, however, the length of the intestine was not shortened at early or at mature stages. In contrast, a slight but statistically significant increase in length was measured for both mutant strains when related to the total body length or body weight [Fig. 1E-G for newborn pups (P0.5) and Fig. S6A-C for post-weaning stages]. All compartments of the intestine were present in CLMP mutants. The overall appearance of the bowel of the mutant was indistinguishable from wild type, except for a severe dilation and for the cecum, which appeared smaller (Figs 1H and 2A-C).

Because malrotation of the intestine – a broad term that encompasses a number of rotational and fixation abnormalities of the intestines – is another hallmark in several case reports of SBS, we monitored the arrangement of the compartments of the intestine in the abdomen of CLMP-deficient mice. Several abnormalities, including a mis-positioning of the cecum and the duodenum, were observed at different stages of maturity in CLMP-deficient mice (Fig. 1H).

### CLMP-deficient intestines revealed reduced amplitudes of pendular movements and a severe dilation

Peristalsis is a coordinated motor behavior that enables the intestine to mix and propel its intraluminal contents to allow efficient digestion of chyme, progressive absorption of nutrients and evacuation of residues. To analyze sinusoidal oscillation (amplitude and frequency), short segments of intact duodenum or jejunum of wild type, heterozygotes or CLMP knockouts fixed with tungsten needles in an organ bath in Krebs-Ringer solution at 37°C were video imaged. To determine the movement longitudinally, and perpendicularly to the longitudinal direction, bifurcation points of blood vessels on the intestine as indicated in Fig. 1I were used for tracking. In duodenum, there was a 6.5-fold decrease (average amplitude in wild type 0.347±0.033 mm and in −/− 0.053±0.005 mm for longitudinal movements) and, in the jejunum, a 17-fold decrease (average amplitude in wild type 0.549±0.055 mm and in −/− 0.0322±0.005 mm) for the amplitude of movement in CLMP-deficient tissue if compared to wild type or heterozygous (Fig. 1J-L,N and Movies 1 and 2). In contrast, the frequencies of motion in both directions were identical between genotypes (Fig. 1M,O).

Analysis of color-coded spatial-temporal maps of gut diameter along the length of an isolated duodenal segment in an organ bath showed a severe dilated situation at zero intraluminal pressure in the knockout in comparison to wild type. The average diameter measured 0.2613 and 0.4102 mm in the wild type and in the knockout, respectively (1.6-fold increase). The change in contraction from maximum to minimum amounted to 38 and 48% of the duodenal diameter in the control and knockout, respectively (Fig. 2A,C). Again, the frequency of contraction was similar between both genotypes, but knockout tissue never contracted as narrow as wild types or heterozygotes. Consistently, measurement of mechanical activity of thin ring preparations of the duodenum tied to hooks of a force transducer and set at resting tension from *Clmp*^−/−^ mice also showed regular spontaneous contractions with a similar frequency and intensity (Fig. 2D), indicating that contraction *per se* is not impaired in the absence of CLMP.

Taken together, our data above suggest that the decreased motility and severe dilation of the small intestine might explain the malnutrition and a severe delay to thrive of CLMP-deficient mice. Consistently, a delayed transport of milk from the stomach into and along the intestine in mutant newborn mice in comparison to wild-type litters was observed. Whereas, in wild-type mice, milk was carried already along the intestine at P0.5, milk was transported only a short distance beyond the stomach in mutant mice (Fig. 2F and images in Fig. 1E). On the other hand, whitely filled stomachs were detected for all inspected P0.5 and P1 mutant mice (Fig. 2E and images from the gastrointestinal tract in Fig. 1E), indicating that milk uptake into the stomach is normal and that a lack of suction is not the cause for the failure to thrive of CLMP-deficient mice. Furthermore, the wet weight of intestine and stomach increased by almost 50% in mature *Clmp* mutants in comparison to wild-type littermates if related to the body weight. Since no further anatomical abnormality was observed in the intestine (see below), the increased wet weight might result from a loaded or rather less emptied gastrointestinal tract (Table S2).

### CLMP-deficient mice develop bilateral hydronephrosis caused by an impaired ureteral motility

**Fig. 3.**
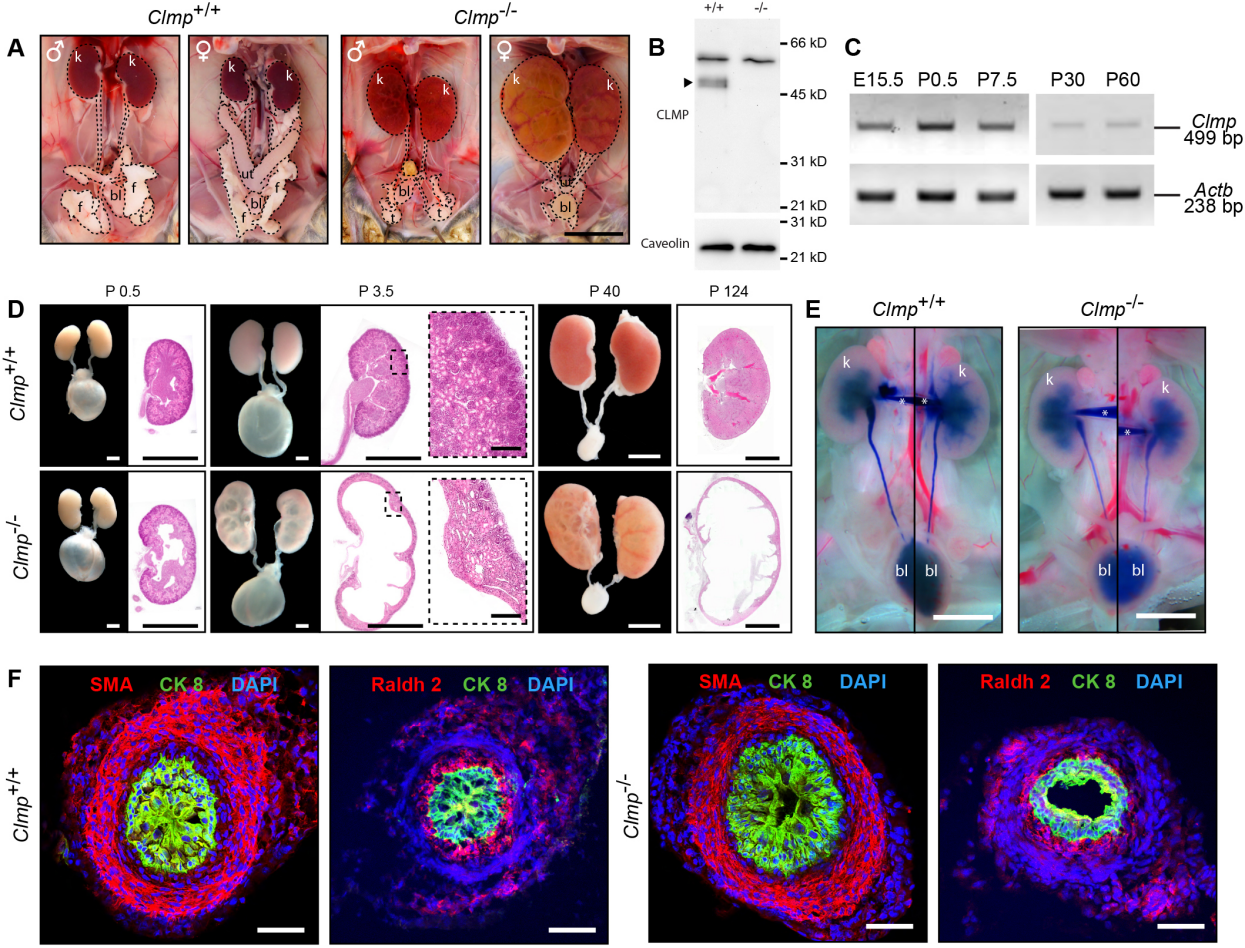
**Bilateral hydronephrosis** **in the absence of CLMP.** (A) Severe degrees of bilateral hydronephrosis and hypomorphic reproductive tracts can be observed in P40 (old) homozygous mutant mice of both genders (mixed genetic background). bl, urinary bladder; f, fat; k, kidney; ut, uterus; t, testes. Scale bar: 1 cm. (B) Absence of CLMP in crude membrane fractions from ureter tissue of 12-week-old mice. CLMP was detected as a doublet at 47 and 48 kDa (arrowhead) using antibody 6504 to the cytoplasmic domain of CLMP. An unspecific band at 60 kDa is observed in both genotypes. Caveolin served as loading control (lower panel). Molecular mass markers are given at the right of the panels. Tissues from five animals of each genotype were pooled. (C) RT-PCR to detect *Clmp* mRNA in ureteral tissue at different stages. *Actb* served as control (lower panel). (D) Bilateral hydronephrosis develops perinatally and aggravates rapidly by dilation of the renal pelvis and loss of renal parenchyma. Whole-mount preparations and HE-stained glycol methacrylate sections are shown. Scale bars P0.5 and P3.5: 1 mm; enlarged image of P3.5: 100 µm; P40 and P124: 5 mm. (E) Intrapelvic ink injections demonstrated the absence of physical obstructions in E18 wild-type (left image) and *Clmp* knockout (right image) urinary systems. *Denotes the pulled micropipette. Scale bars: 2 mm. (F) Immunofluorescent stainings with the smooth muscle marker α-SMA, urothelial marker cytokeratin 8 (CK8) and the ureteral stromal marker retinaldehyde dehydrogenase (Raldh2) show normal structure of ureteral cell layers in CLMP-deficient P1.5 mice. Scale bars: 50 µm.

In addition to the impaired intestinal motility, mature CLMP-deficient mice develop severe bilateral hydronephrosis, which involves dilatation and distension of the renal pelvis and calyces (Fig. 3A,D). Consistently, the wet weight/body weight ratio of the kidney from mature mutants on average increased approximately 4-fold (Table S2). Hydronephrosis became already apparent at early postnatal stages, but markedly increased with age (Fig. 3D). The concentrations of ions, urea, creatinine or total protein in the urine of mature CLMP-deficient mice was not altered (Fig. S7A). However, western blotting revealed an increased level of urinary NGAL (neutrophil gelatinase-associated lipocalin; also termed lipocalin2) (Mori and Nakao, 2007; Paragas et al., 2011), a marker of renal tubular injury, in CLMP-deficient mice already at early postnatal stages (Fig. S7B). This suggests that hydronephrosis caused tissue damage to the renal parenchymal tissue.

**Fig. 4.**
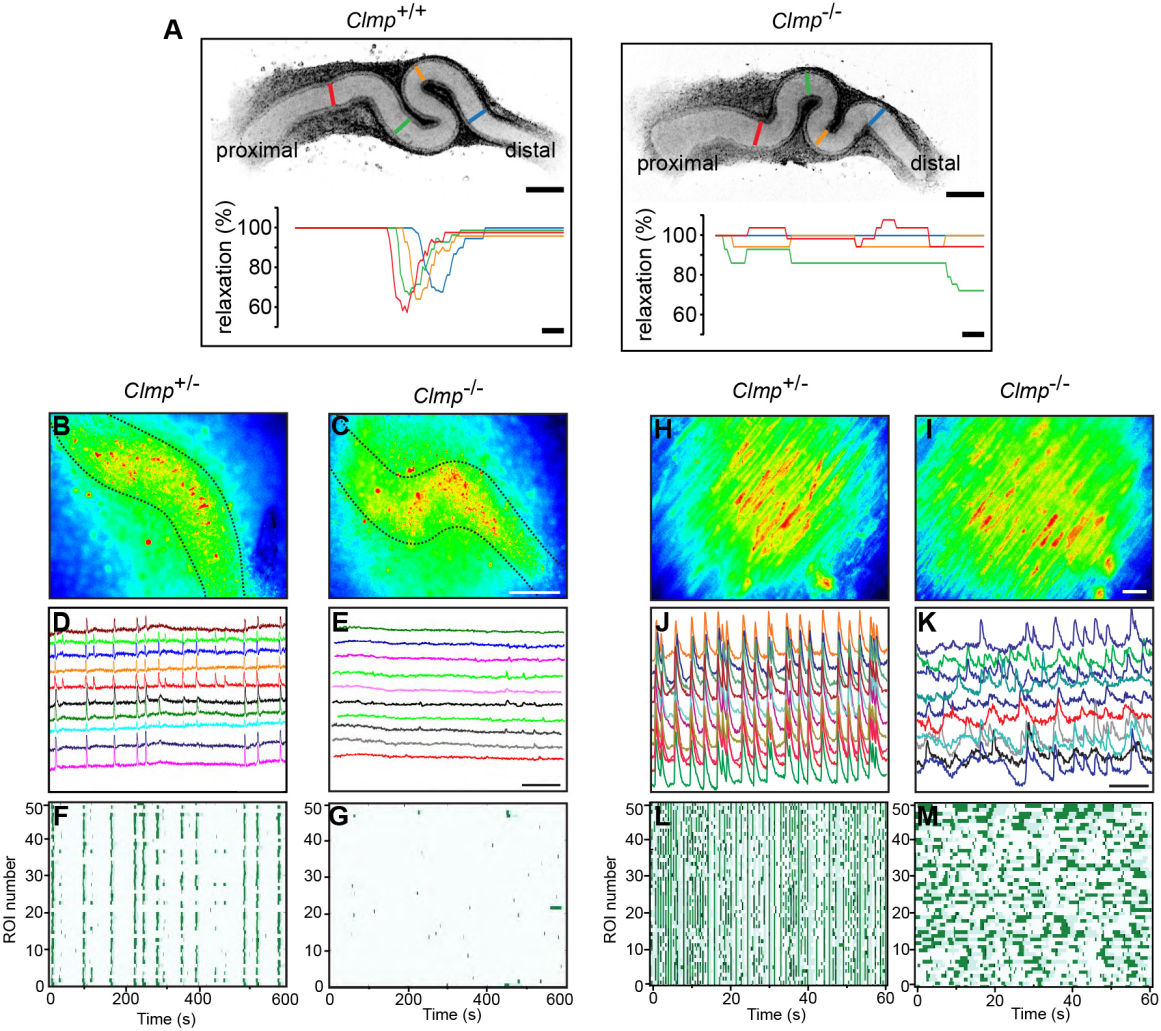
**Impaired peristaltic ureteral motility and uncorrelated calcium transients indicate impaired cell-cell communication in smooth muscle cell layers of intestine and ureter.** (A) *Ex vivo* culture of wild-type ureteral explants (left panel) displayed ureteral peristaltic contractions, whereas ureters lacking CLMP (right panel) failed to develop peristalsis. Time-lapse recordings (270 ms intervals) of ureters were taken from wild-type and CLMP-deficient E15 embryos after 5 days *in vitro* (Movies 3, 4). The images reveal peristaltic contraction waves in control ureters, whereas mutant ureters display uncoordinated fibrillation-like movements that did not constrict the lumen diameter strongly. A single peristaltic wave is plotted as percent relaxation of the ureteral lumen diameter at the respective sites as indicated by colors. Contraction intensities were comparable to published data (Bush et al., 2006). In total 26 knockout, 10 wild-type and 16 heterozygote ureter were analyzed with similar results. Scale bars upper images: 200 µm; lower panels: 2 s. (B-G) Calcium imaging of ureteral explants of heterozygotes or CLMP-deficient mice were loaded with Fura-2 at DIV 5 (wild type *n*=3, heterozygotes *n*=5, knockout *n*=4). (B,C) Fluorescence images of Fura-2-loaded ureteral explants. Scale bar: 100 µm. (D,E) Single traces of 10 selected regions of interest (ROIs) are shown. Scale bar: 100 s. (F,G) Raster plots of 50 simultaneously recorded ROIs. (H,I) Fluorescence images of Fura-2-loaded duodenal strips. The smooth muscle cell layer is oriented upside and villi down. Scale bar: 20 µm. (J,K) Single traces of 10 selected ROIs are shown from longitudinal smooth muscle cells. Scale bar: 5 s. (L,M) Raster plots of 50 simultaneously recorded ROIs. Twelve strips from four animals aged 6-8 weeks were analyzed.

Hydronephrosis might result from multiple etiological factors, including physical or functional blockage in the ureteral system. On grounds of our observations on the intestine, we studied a functional obstruction in the ureter by culturing ureters from embryos of CLMP-deficient and wild-type mice. *Clmp* mRNA is expressed in the embryonic and mature ureter, and CLMP protein was detected by western blotting in mature ureter (Fig. 3B,C and Fig. S3B). Ureters have been shown to exhibit peristalsis independently from the renal pelvis in culture (Cain et al., 2011). Cultured wild-type embryonic ureters started unidirectionally coordinated contractions from the proximal to the distal part after 4 days of *ex vivo* organ culture. After 5 days of culture, control ureters showed approximately 0.4 contractions per minute. In contrast, CLMP-deficient ureters were completely unable to form proximal-to-distal directed peristaltic waves. Even after extended cultivation periods, ureters of *Clmp* knockout mice were not capable of developing contraction waves. In the absence of CLMP, predominantly uncoordinated, fibrillation-like movements of the ureter wall without full contractions (Fig. 4A and supplemental time-lapse Movies 3 and 4) were detected. It is important to note that ureteral explants from *Clmp*^−/−^ mice after 5 days of cultivation did not differ with respect to length from wild-type and heterozygous tissue [wild type, 2443±125 µm (*n*=10); heterozygous, 2193±97 µm (*n*=12); knockout, 2451±100 µm (*n*=13)].

To exclude any physical blockade, we analyzed liquid flow from the renal pelvis to the bladder by intrapelvic ink-injection experiments. In wild type and *Clmp*^−/−^ mutants, the ink easily flowed through the urinary path to the bladder; hence, physical blockage along the ureter did not contribute to the hydronephrotic phenotype in *Clmp* mutants (Fig. 3E). *Clmp* knockout mice also did not reveal renal malformations or shortened ureters.

Conventional histology and immunohistochemistry indicated that the structure of the kidney as well as the overall organization of the urinary tract is not affected at early postnatal stages. Immunofluorescent stainings for smooth muscle actin, Raldh2 or cytokeratin 8 revealed a normal layer architecture of the ureter with smooth muscle and epithelial layers in *Clmp-*null ureters (Fig. 3F). These data suggest that loss of the cell adhesion molecule CLMP does not lead to overall developmental deficits or to a distortion of tissue architecture in the ureter.

Thus, hydronephrosis develops in *Clmp* mutants from the functional urinary tract obstruction of urine transport in the absence of any structural abnormalities within the ureter. In this respect, the ureteral defects are similar to the lack of an effective transport of chyme in the CLMP-deficient intestine. The bilateral ureteral obstruction might also contribute to a premature death of mutant mice, in particular at advanced stages.

### Calcium transients in smooth muscle cell layers of intestine and ureter are uncorrelated in the absence of CLMP

To further understand the deficits in motility in the intestinal and ureteral system, we analyzed calcium signal propagation in smooth muscle cells of ureter and intestine. Ureter explants after 5 days *in vitro* were loaded with the dye Fura-2 followed by calcium imaging. Calcium signals were measured at 50 equally spaced regions of interest (ROIs) in each explant. In wild-type explants, correlated calcium waves were detected, whereas, in mutants, no coordination between calcium signals was observed. Mutant ureteral explants exhibited only few and weak calcium transients (Fig. 4B-G). In duodenal strips from wild-type postnatal stages, highly correlated calcium transients were observed in the longitudinal smooth muscle cell layer. In contrast, in CLMP-deficient duodenal tissue, only uncoordinated events were measured (Fig. 4H-M).

### Neurotransmission was not impaired in CLMP-deficient mice

**Fig. 5.**
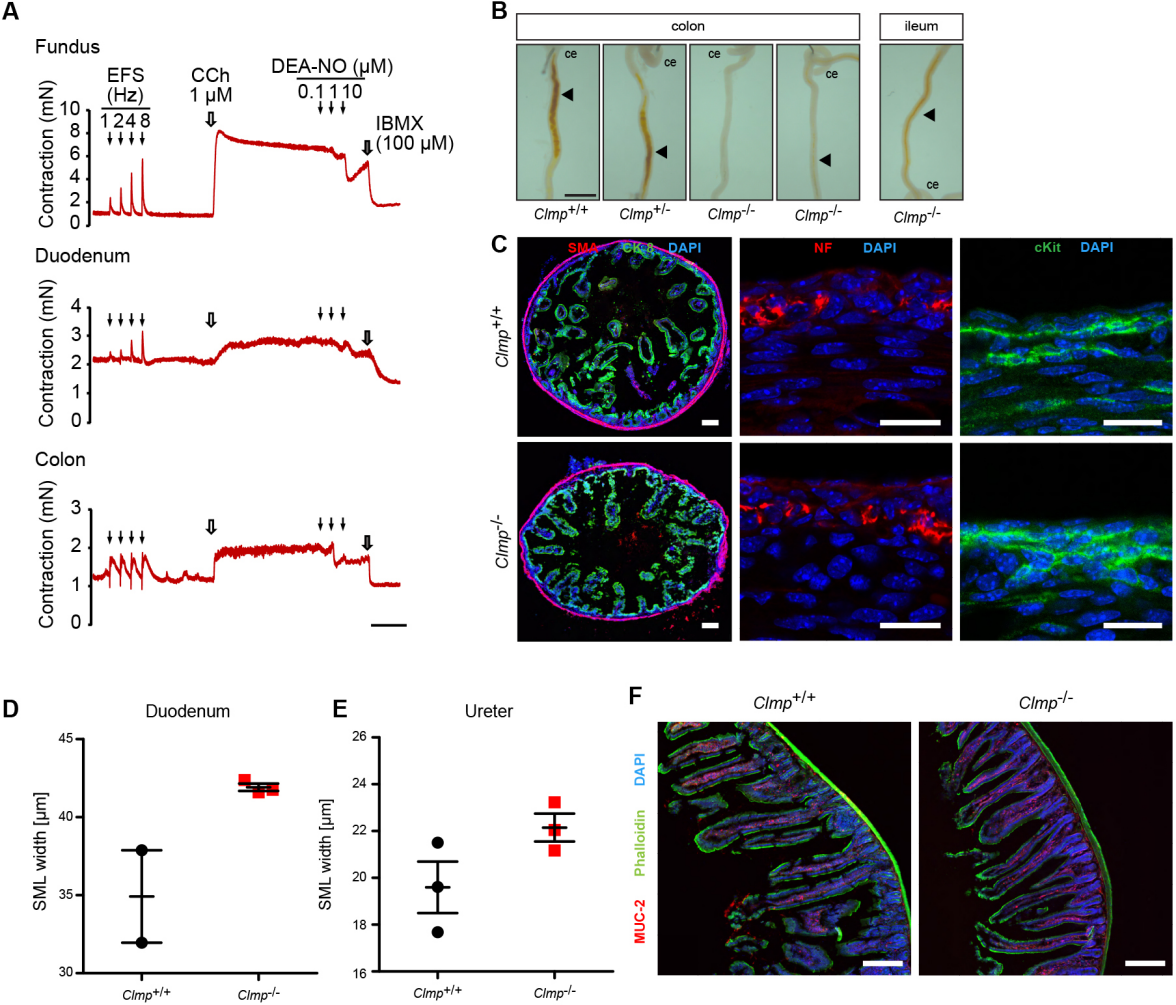
**The transport of meconium is delayed, the neurotransmission within the gastrointestinal tract is not impaired and the thickness of the smooth muscle layer is slightly increased in CLMP-deficient mice.** (A) Fundus strips or rings from duodenum or colon from P4 *Clmp*^−/−^ mice were mounted in an organ bath with a resting tension of 1 mN. Electric field stimulation (EFS) was applied at 1, 2, 4 and 8 Hz inducing a response from various released transmitters, including acetylcholine and NO. Then, tissues were pre-contracted with CCh (1 µmol/l) followed by relaxation using increasing concentrations of DEA-NO followed by IBMX (100 µmol/l). Shown are representative traces. Similar results were obtained from P2 tissue. Scale bar: 10 min. (B) Meconium (arrowhead) was completely absent or found in tiny amounts in the colon at E18.5 in CLMP-deficient mutants, in contrast to wild type. Meconium is occasionally detected in the mutant ileum (right panel). In total 4 knockouts and 7 wild types or heterozygotes were inspected. ce, cecum. Scale bar: 20 mm. (C) Immunofluorescent stainings of cross-sections with antibodies to smooth muscle actin (SMA), cytokeratin 8 (CK 8), neurofilament (NF) or c-Kit do not reveal differences in the duodenum of P1.5 wild-type and CLMP-deficient mice. Scale bars in the left panels: 100 µm; in the middle and right panels: 20 µm. (D,E) The cross-sectional thickness of the smooth muscle layer (SML) of the intestine and ureter showed a tendency to be increased. Cross-sections of ureter or duodenum were stained by an antibody to smooth muscle actin and the width was measured in microscopic images. (See also Fig. 7A and B; data are shown as means±s.e.m., *t*-test, *P*=0.051 in D and *P*=0.111 in E.) (F) Cross-sections of the duodenum (4 weeks old) stained by phalloidin, DAPI and an antibody to mucin 2. The latter stains Goblet cells. The pattern of localization of Goblet cells was indistinguishable between wild-type and *Clmp* knockout intestine. Scale bars: 200 µm.

Movement of intestinal contents requires contraction/relaxation of longitudinal and circular smooth muscle layers, which is regulated by an interplay between the enteric neuronal network, interstitial cells of Cajal (ICC) and intrinsic mechanisms of smooth muscle cells (Bornstein et al., 2004; Farrugia, 2008; Huizinga and Lammers, 2009; Sanders, 2008). To study the capability of the smooth muscle cells to respond to endogenously released contractile agonists, we performed electric field stimulation (EFS) of ring or strip preparations of gastrointestinal tissues mounted in an organ bath. EFS elicited normal contraction in rings from duodenum or colon as well as in fundus strips from P4 *Clmp*^−/−^ mice (Fig. 5A). In addition, all three tissues contracted upon carbachol (CCh) administration (1 µmol/l) and relaxed after addition of the guanylyl cyclase activator DEA-NO, a nitric oxide (NO) donor, or the phosphodiesterase blocker IBMX (3-isobutyl-1-methylxanthine), demonstrating functional neurotransmission via acetylcholine receptors and NO-sensitive guanylyl cyclase.

Consistently, a severely delayed passage of meconium in the intestine was observed. Whereas, in wild types and heterozygotes, it was located in the colon at embryonic day 18.5, as described by others, in mutants it reached only the ileum, suggesting a failure of propulsion of the meconium (Fig. 5B). This progression of contents through the embryonic gut does not require the activity of enteric neurons (Anderson et al., 2004).

Stainings of transversal duodenal sections from P0.5 mice against neurofilament, c-Kit or cytokeratin 8 showed a normal overall pattern of cell layers, including the localization of enteric neurons and of ICC and a normal intestine lumen diameter (Fig. 5C). The latter excluded physical barriers in the intestine. In contrast, the smooth muscle cell layer of the intestine and ureter appeared thicker in cross-sections from *Clmp* mutants, although this did not reach statistical significance if quantified (Fig. 5D,E). In addition, Goblet cells, which have been shown to be absent in a *clmp* zebrafish morpholino knockdown (Van Der Werf et al., 2012), are present in CLMP knockout mice and their pattern of localization is indistinguishable from that of wild-type intestines (Fig. 5F).

In addition to the lack of coordinated contraction of the intestine, physical barriers or other deficits in upper parts of the gastrointestinal tract might provoke a reduced transit of nutrients. For example, human SBS is often accompanied by a hypertrophic pyloric sphincter (PS) (Van Der Werf et al., 2015; Schalamon et al., 1999), and the lower esophageal sphincter (LES) as well as fundus might be contemplable for an impaired milk transport from the stomach into the duodenum. The composition of cellular layers in the PS, including smooth muscle cells and c-Kit-positive cells, in *Clmp* mutants were indistinguishable from wild-type littermates (Fig. S8A-C). A narrowing of the pyloric opening that might impede the passage of food was not detected in longitudinal sections excluding a pyloric stenosis (Fig. S8B). However, an elongated shape of the transition zone from the antrum to the duodenum (Fig. S8A) was observed in the majority of preparations.

These anatomical data are in line with observations on contractions of the PS, fundus and the LES. Individual thin ring preparations of PSs exhibited spontaneous rhythmic contractions when mounted in an organ bath. Spontaneous contraction frequency was not different between *Clmp*^+/+^ and *Clmp*^−/−^ mice but, unexpectedly, the maximal contraction force was significantly increased in the knockout, indicating a higher muscle tone (Fig. S8D-I). CCh, a muscarinic agonist, was used to pre-contract fundus strips and rings of the LES. For both, the concentration response curve to the NO donator DEA-NO was shifted to higher values in *Clmp*^−/−^, indicating a reduced sensitivity towards NO and a higher muscle tone (Fig. S8D-I). These data indicate the ability of smooth muscle cells in the gastrointestinal tract to contract; moreover, they demonstrate an increased tone of gastrointestinal smooth muscle.

In summary, lack of effective chyme transport within the intestine in the absence of CLMP may originate from a peristaltic dysfunction.

### CLMP is essential for expression of Connexin43 and 45 in the intestinal, and of Connexin43 in the ureteral, smooth muscle layer

**Fig. 6.**
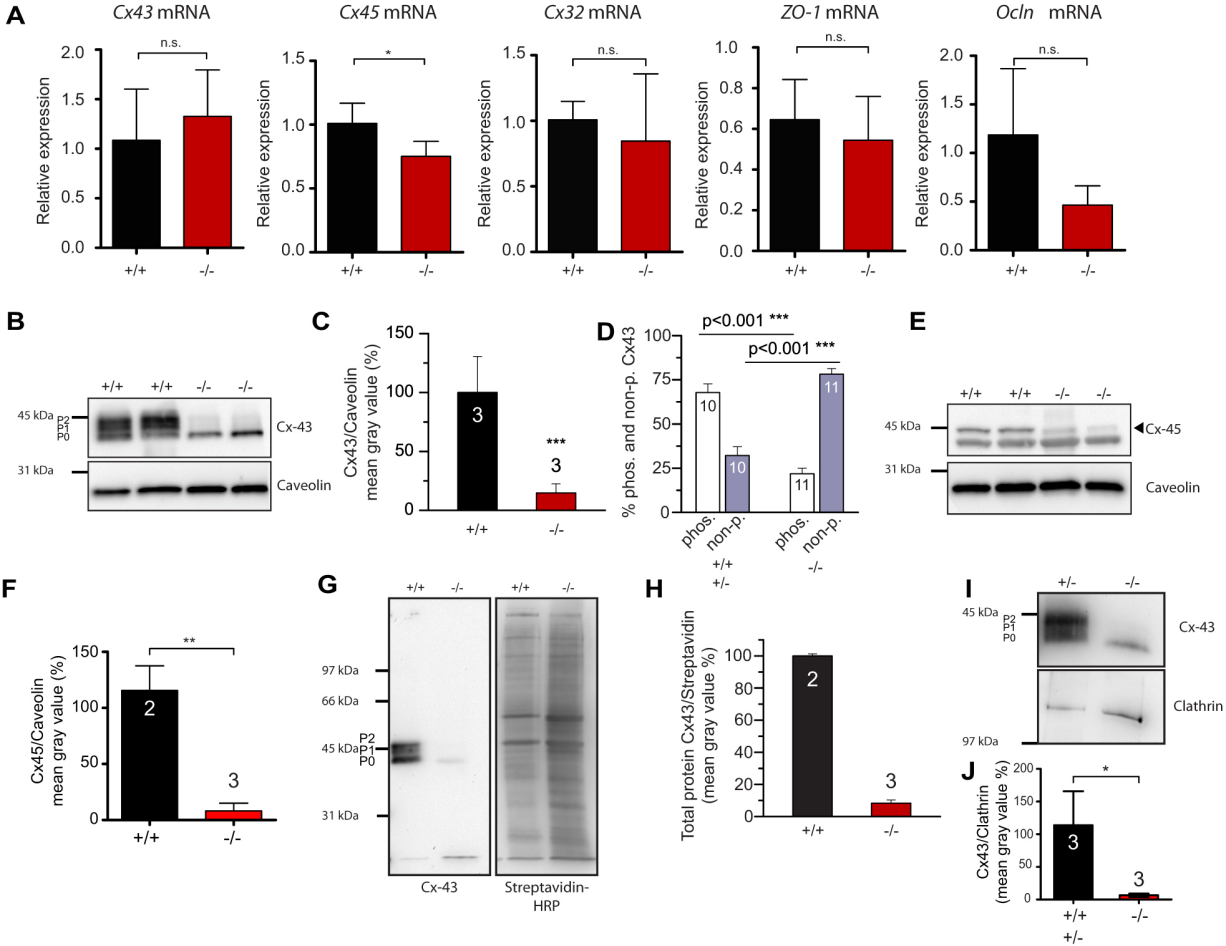
**Expression of Connexin43 and 45 is decreased in the intestinal smooth muscle cell layer and Connexin43 in the ureter in the absence of CLMP.** (A) qRT-PCR of Connexin43, 45, 32, *ZO-1* and occludin mRNAs reveal a slight downregulation only for Connexin45. *Rplp0* served as housekeeping gene. (Data are shown as means±s.d., *Cx43* *P*=0.51, *n*=4 per genotype; *Cx45* *P*=0.039, *n*=4 per genotype; *Cx32* *P*=0.45, *n*=4 per genotype; *ZO-1* *P*=0.55, *n*=3 for +/+ and *n*=4 for −/−; *Ocln* *P*=0.09, *n*=3 for +/+ and *n*=4 for −/−; *P≤0.05). (B,C) Connexin43 is strongly reduced in the smooth muscle layer of the intestine in the absence of CLMP. Anti-caveolin served as loading control. P0 indicates unphosphorylated Connexin43 and P1 and P2 phosphorylated forms. (D) Phosphorylation of Connexin43 in the smooth muscle layer is reduced in *Clmp* mutants. Numbers in columns indicate number of blots analyzed. The ratio of phosphorylated to non-phosphorylation was calculated. (E,F) Connexin45 (arrowhead) expression is reduced in the smooth muscle cell layer of the intestine in *Clmp* mutants. Connexin45 levels were related to caveolin. Please note, a most likely unspecific band at 40 kDa is detected by the antibody to Connexin45 in intestinal smooth muscle cell layer of both genotypes. (Data shown as means±s.d., **P=0.0035, *n*=2 for +/+ and *n*=3 for −/−.) (G,H) Enrichment of cell surface proteins from the smooth muscle layer of the intestine by the Sulfo-NHS-biotin method followed by western blotting shows that Connexin43 is only very weakly expressed on the surface of smooth muscle cells. The right panel shows the labeling of the same blot with streptavidin-HRP to indicate loading. Surface expression of Connexin43 was related to total protein as detected by streptavidin-HRP. (I,J) Expression of Connexin43 in ureter is strongly reduced in *Clmp* knockouts. Anti-clathrin served as loading control. Please note that total ureter was blotted due to the low amount of tissue. Fat tissue could not be completely removed from the ureter. Molecular mass markers are indicated at the left of each panel showing western blots. (J) The level of Connexin43 in the ureteral tissue reached 5.7% of control values (*P=0.023).

Overall, our data indicate an impaired and uncoordinated motility and cell-cell communication in the absence of CLMP in ureteral and intestinal smooth muscle cells, which form a functional syncytium mainly by gap junctions to synchronize events (Maes et al., 2015). Therefore, we analyzed the pattern of expression of Connexin43 – the major connexin in the circular muscle layer of the intestine – Connexin45 and Connexin32, which are also expressed in the smooth muscle layer (Willecke et al., 2002), and the junctional components ZO-1 and occludin. In qRT-PCR, the level of Connexin43-, Connexin32-, ZO-1- and occludin-encoding mRNAs were not altered, whereas that of Connexin45 was slightly reduced in the smooth muscle layer of the intestine (Fig. 6A). In contrast, western blot analysis revealed a drastically decreased level of protein expression for Connexin43 and 45 (Fig. 6B,C and E,F). The level of protein expression in the smooth muscle cell layer of the intestine of CLMP-deficient mice reached only 15% of control levels for Connexin43 and 8% for Connexin45. Furthermore, an increased electrophoretic motility of Connexin43 was observed from CLMP-deficient mice in comparison to wild-type tissue. Most of the protein migrated at a position that represents unphosphorylated Connexin43 (P0 form, 78%), whereas, in wild-type smooth muscle, Connexin43 exists mainly as a phosphorylated form (P1 and P2 forms, 67.8%) (Fig. 6B,D). Phosphorylation of Connexin43 has been implicated in the regulation of gap junctional communication at different levels and in trafficking (Solan and Lampe, 2016; Falk et al., 2016; van Veen et al., 2000). Therefore, cell surface proteins were enriched from the smooth muscle layer of the intestine by the Sulfo-NHS-biotin labeling method. Western blotting revealed an expression for Connexin43 below 10% of wild-type values in this fraction (Fig. 6G,H). A decreased expression and phosphorylation pattern of Connexin43 was also detected in ureter knockout tissue (Fig. 6I,J), where the total protein level reached 5.7% of control values.

**Fig. 7.**
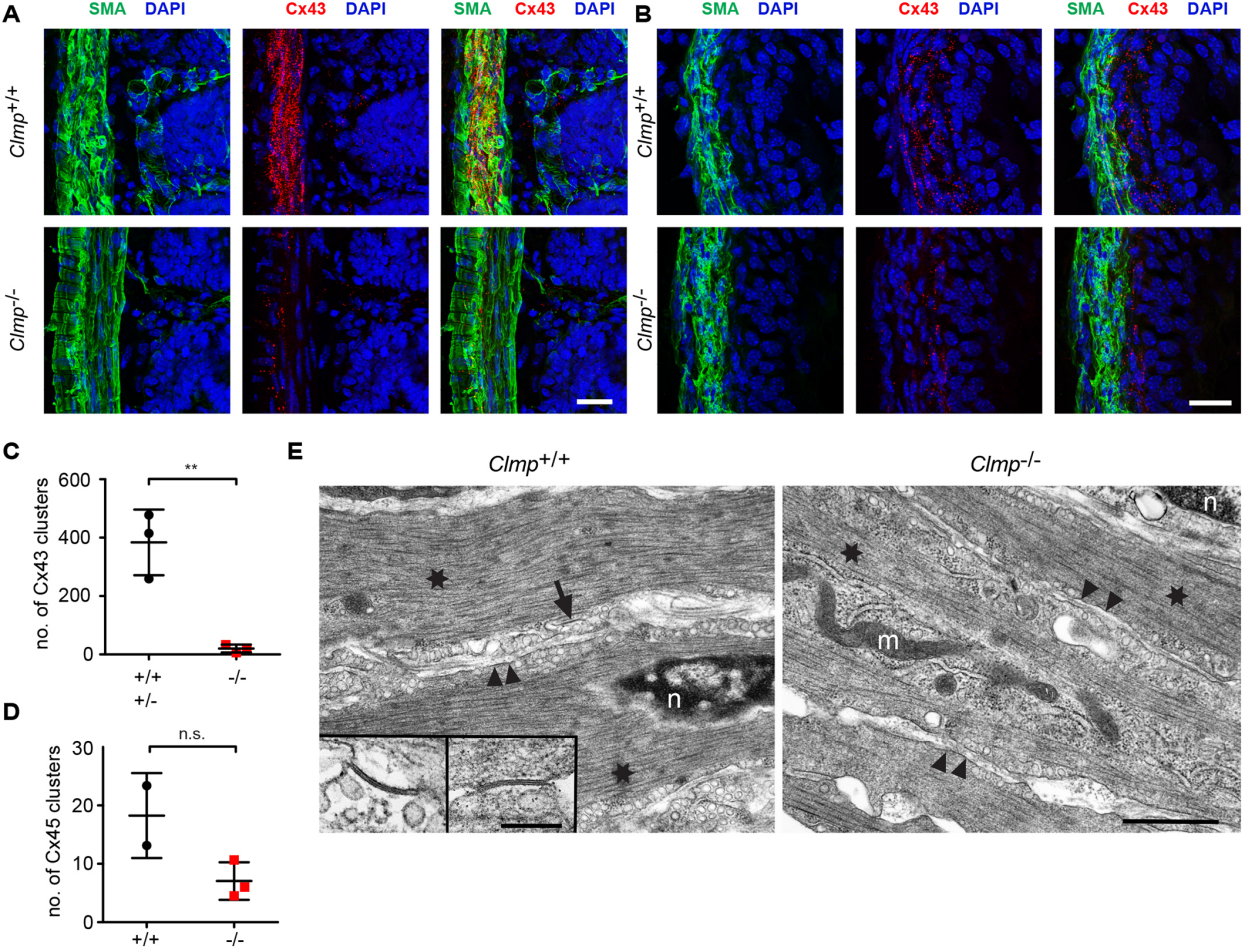
**Connexin43 clusters are strongly reduced in CLMP-deficient duodenum and ureter.** (A,B) Connexin43 plaques are reduced in the circular smooth muscle cell layer of CLMP-deficient duodenum (A) and smooth muscle layer of the ureter (B). In the ureter, Connexin43 spots reached 21.9% of wild-type values. Scale bars: 20 µm. (C,D) The quantification of Connexin43 and Connexin45 spots in the duodenum. (Data shown as means±s.d. Cx43 cluster, *P*=0.0051, *n*=3 per genotype; Cx45 cluster, *P*=0.089, *n*=2 for +/+ and *n*=3 for −/−). Stainings for Connexin45 in the intestine are shown in Fig. S9. (E) Ultrastructure of circular smooth muscle cells in the duodenum of wild-type and CLMP-deficient mice. No differences were observed in the arrangement of actin-myosin filaments. Gap junctions were only detected in wild types, as indicated at higher magnifications in inserts. In total, about 20,000 µm^2^ and 50,000 µm^2^, representing roughly 500 cells and 1250 cells, in wild-type and knockout tissue, respectively, were inspected in 70-nm-thick sections. Scale bars: 1 µm and 200 nm (insert). n, nucleus; m, mitochondria; arrow, gap junction; arrowheads, plasma membrane; stars, actin-myosin filaments.

In tissue sections, a strongly clustered localization of Connexin43, most likely representing membrane-localized gap junctional plaques that contain thousands of intercellular channels, was detected in wild-type circular smooth muscle layer, whereas, in knockout tissue, we observed a severely decreased number of Connexin43 puncta in intestine (Fig. 7A,C) and of Connexin43 in ureter (Fig. 7B). Similarly, a decrease of Connexin45 staining was observed in the smooth muscle layer of the intestine (Fig. 7D and Fig. S9). Electron microscopic images revealed a normal ultrastructure of smooth muscle cells but a complete absence of gap junctions in the circular smooth muscle layer of CLMP-deficient intestine. Annular gap junction vesicles representing degrading gap junctions (Falk et al., 2016) were not detected in CLMP-deficient intestine (Fig. 7E).

Together, the biochemical and histological data indicated a decreased expression of Connexin43 and 45 in intestinal and ureteral knockout smooth muscle cells, which in turn impaired cell-cell communication and consequently coordinated motility.

## DISCUSSION

In the present study, we observed that the homophilic IgCAM CLMP accomplishes specific functions in peristalsis by regulating cell surface expression on smooth muscle cells of Connexin43 and 45 in the intestine and Connexin45 in the ureter. Our biochemical and histological investigations demonstrated a reduced expression of Connexin43 and 45 in the CLMP-deficient smooth muscle cell layer (in the range of 10% of control values). In this regard the *Clmp* mutant might be considered as an – although incomplete – double mutant for Connexin43 and 45 in smooth muscle cells of the intestine, which also might explain in part the more severe intestinal phenotype in *Clmp* mutants than that described for Connexin43 alone (Döring et al., 2007). The presence of CLMP appears to be a prerequisite for the formation of Connexin43- and/or Connexin45-containing gap junctions in intestinal and Connexin43-containing junctions in ureteral smooth muscle cell layers. The level of mRNAs encoding Connexin43 is not and that of Connexin45 is only slightly reduced in the intestine of knockout mice compared with wild type, suggesting that the turnover rate of Connexin43 and 45 might be shifted towards an increased degradation or that translational interferences might lower their expression in CLMP-deficient smooth muscle cells. Connexins in general have a very short half-life of only 1-5 h and for Connexin43 it is well known that phosphorylation at specific sites by kinase signaling pathways, including Akt, mitogen activated protein kinase (MAPK), PKC or Src, controls gap junction assembly, size, disassembly and degradation (Solan and Lampe, 2014, 2016; Falk et al., 2016).

The severe reduction of gap junctional communication by the global deletion of *Clmp* in the mouse resulted in impaired peristaltic transport processes in the intestine and ureter. Consistently, analysis of calcium transients in the smooth muscle cell layer of the ureter or intestine indicated an impaired cell-cell communication in the absence of CLMP. This cell layer of the intestine or ureter consists of smooth muscle cells in series and in parallel forming an electrical syncytium that facilitates synchronous excitation of a large number of cells to permit coordinated activity. Previously published electron microscopic investigations of the intestine revealed gap junctions between circular smooth muscle cells which are mainly composed by Connexin43 as determined by immunohistological methods (Daniel and Wang, 1999; Mikkelsen et al., 1993; Seki and Komuro, 2001; Wang and Daniel, 2001), whereas the intercellular communication in the longitudinal smooth muscle layer of the intestine is less understood and might be devoid of gap junctions (Farraway et al., 1995).

The overall phenotype of *Clmp* knockout mice partly mirrors the situation in CSBS patients with mutations in the human *CLMP* gene, including malnutrition, lack of gain of body weight, intestinal malrotation and a high mortality rate at early postnatal stages. A major discrepancy between human patients and *Clmp* mouse mutants is the length of the intestine compared with their wild-type counterparts. Whereas, in human CSBS patients, the small bowel fails to elongate, CLMP-deficient mice displayed a normal length of the intestine; in fact, when the body length or body weight is taken into account, an even slightly longer intestine was measured. The distinction in gut length might reflect different developmental processes in the length growth of the intestine in humans and mice. For example, impaired peristaltic contractions or the gestational status has been discussed to correlate with the process of elongation in humans (Schalamon et al., 1999; Van Der Werf et al., 2015). Alternatively, compensatory mechanisms might exist in the mouse that affect the elongation of the intestine. In general, absence of CLMP appears to be more severe in humans in comparison to mice since only 22% of the patients survived more than 1 year. Shortening of the intestine might be the responsible factor for this graveness. It is highly likely that the major cause of premature death of CLMP-deficient mice is the impairment of coordinated waves of contractions in the gastrointestinal tract, which are essential for an effective transport and adsorption of nutrients. We did not observe any physical obstructions. Furthermore, spontaneous as well as ligand- and EFS-induced contractions of ring preparations appeared normal in the gastrointestinal tract, whereas the coordinated contraction of larger segments of the intestine was severely impaired, which is in agreement with the observed reduced transport of milk and meconium. The increased muscle tone of pyloric tissue and the slight increase in thickness of the smooth muscle layer might be considered as a compensatory mechanism due to a failure of propulsive transport in the intestine and ureter.

In addition to the intestinal deficits, CLMP-deficient mice that survived past weaning age developed a severe bilateral hydronephrosis that has not been described in human CSBS patients or might have been overlooked due to their early lethality. However, recently two siblings with *CLMP* mutations have been described with intestinal dysmotility and, in addition, ureteropelvic junction obstruction (Alves et al., 2016). It is conceivable that this obstruction might result from impaired peristalsis of the ureter. Hydronephrosis might be caused by an impaired transport of urine from the kidney to the bladder. After filling of the renal pelvis with urine, unidirectional peristaltic contractions are initiated at the renal pelvis and are propagated via the ureter, propelling urine from the pelvis of the kidney towards the urinary bladder. A failure of this action causes flow impairment leading to urinary tract dilation, hydronephrosis and finally kidney damage. Our anatomical studies as well as intrapelvic ink injections did not provide evidence for physical obstructions or malformations in CLMP-deficient ureters. In contrast, our data demonstrated a functional obstruction of the ureter as found for the intestine. Consequently, cultivations of ureters indicated an impaired peristalsis. Hydronephrosis in CLMP-deficient mice is therefore likely a secondary consequence of the lack of urine transport and back pressure from the fluid-filled ureter.

Several types of enteric neurons in the gastrointestinal tract have been detected and intestinal motility depends on the coordinated activity of peripheral neurons (Bornstein et al., 2004; Sanders, 2008). In contrast, tetrodotoxin has little effect on the peristalsis of the ureter, suggesting that the peripheral nervous system plays a negligible role in maintaining pyeloureteral motility (Lang et al., 2002, 2001). Assuming that ureteral and intestinal malfunctions have an identical molecular basis in CLMP deficiency, it is plausible to hypothesize that the impaired transport is not caused by deficits in the neuronal organization or communication in the intestine. This interpretation is also supported by our physiological and histological studies on the gastrointestinal tract, which did not show any neuronal deficits. Furthermore, the failure of the meconium to pass through the intestine at late embryonic stages supports our view that a neuronal dysfunction is not implicated in the CLMP phenotype.

The intestine is an elongated tubular structure that is folded into a compact structure through the process of looping morphogenesis. All cases of CSBS were combined with a mispositioning of the intestine – termed malrotation – which was also manifested in CLMP-deficient mice (Van Der Werf et al., 2015). During embryonic development, the intestine extends into the coelom of the body and returns into the abdomen accompanied by rotation. It is thought that herniation plays a role during these processes (Van Der Werf et al., 2015; Schalamon et al., 1999). Furthermore, there is evidence that the looping patterns are determined by the differential growth of the gut tube and the dorsal mesentery or by differential adhesion to asymmetric localized extracellular matrix proteins (Savin et al., 2011). The role of the cell adhesion protein CLMP in these processes remains to be determined. It is also likely that the enlarged kidneys in *Clmp* mutants might aggravate mispositioning of intestinal segments.

In sum, our study uncovered a role of CLMP in transport processes of urine to the bladder and of chyme through the gastrointestinal tract by regulating the level of Connexin43 and 45 expression in smooth muscle cells. Therefore, this adhesion molecule is crucial for normal smooth muscle contractility. However, the detailed molecular role of how CLMP affects cell surface localization of Connexin43 or 45 – either by regulating the turnover rate, by interfering with translation or by intracellular trafficking processes – remains to be established. ZO-1 interacts with several gap junction proteins, including Connexin43 and 45 (Giepmans and Moolenaar, 1998; Toyofuku et al., 1998; Laing et al., 2001; Kausalya et al., 2001), and CLMP was found to colocalize with ZO-1 in transfected cells (Falk et al., 2016; Raschperger et al., 2004). Formation of a molecular complex between CLMP and ZO-1 and Connexin43 or Connexin45 is therefore conceivable. Our studies are in part in line with observations on the related IgCAMs CAR and BT-IgSF, which might be implicated in the regulation of Connexin45 or Connexin43 in cardiomyocytes or Sertoli cells, respectively (Lisewski et al., 2008; Lim et al., 2008; Pelz et al., 2017). Furthermore, whether CLMP exerts its function on Connexin43 or 45 also in other cell types such as neurons or smooth muscle cells of the cardiovascular system would be an essential topic of future research. With further baseline characterization of the timing and molecular mechanisms on the progression of ureteral and intestinal defects, further studies might allow the development of strategies to modulate CLMP and in turn Connexin43 and 45 activity. Overall, our data provide further insights for our understanding of obstructive diseases.

## MATERIALS AND METHODS

### Generation of *Clmp* mouse mutants and genotyping

The *Clmp* target vector was generated by Vega BioLab (USA) by standard procedures, replacing exon 1 of the mouse *Clmp* gene with a neomycin cassette. A thymidine cassette was inserted in the 3′ homologous arm of the target vector for negative selection of embryonic stem (ES) cells. Relevant parts of the target vector were verified by sequencing. ES cells E14.1 derived from 192P2/OlaHsd were electroporated and homologous insertion was tested after digestion with *Bgl*II by Southern blotting. Positive ES cells were injected into blastocysts derived from C57BL/6 by the transgenic core facility of the Max-Delbrück-Center (MDC) and chimera that transmitted the mutated *Clmp* gene were identified by mating to C57BL/6 females. Routine genotyping was performed by PCR using oligonucleotides P1 (5′-GAGAACCGTTTCGTGGAGAG-3′) vs P2 (5′-TTCAGGAGGGGCAGAATATG-3′) for amplification of a 459 bp wild-type fragment, and P3 (5′-TCCTAAGAAGGGACGACGAG-3′) vs P4 (5′-AGCCAGTAAGCAGTGGGTTC-3′) for a 428 bp knockout amplicon. Genotypes were verified by Southern blot hybridization. The mutant mice were bred on a mixed SV129/C57BL/6 background (B6;129P2-*Clmp*^tm1a^/Fgr) or backcrossed to a C57BL/6 background (B6.129P2-*Clmp*^tm1a.1^/Fgr) in which the neomycin cassette had been removed by a Cre-deleter strain (129S1-*Hprt*^tm1(cre^^)^/Mnn) (Schwenk et al., 1995).

The animal procedures were performed according to the guidelines from directive 2010/63/EU of the European Parliament on the protection of animals used for scientific purposes. All experiments were approved by the local authorities of Berlin (numbers T0313/97, 0143/07 and G0370/13).

### qPCR to detect *Clmp* mRNA in intestinal tissue

Total RNA from the intestinal smooth muscle cell layer or villi were isolated using the RNeasy Mini Kit (Qiagen), including an on-column DNase I digestion. RNA yield was measured by Nanodrop 1000 (Nanodrop). RNA was transcribed by SuperScript II (Invitrogen) using oligo dT Primers. The qRT-PCR was performed on a 7500 Fast Real-Time PCR System (Applied Biosystems) with GoTaq qPCR Master Mix (Promega). *Rplp0* was used as a housekeeping gene. The following primers (5′-3′) were used: for *Clmp* (intron-flanking exon 6 and 7) forward: CAGGAGCAGTGACAGGCATA; *Clmp* reverse: AGGAGCTAGGCTTCACAAGG; for *Rplp0* forward: GGACCCGAGAAGACCTCCTT; *Rplp0* reverse: GCACATCACTCAGAATTTCAATGG; for *Cx43* forward: GAACACGGCAAGGTGAAGAT; *Cx43* reverse: GACGTGAGAGGAAGCAGTCC; for *Cx45* forward: AAAGAGCAGAGCCAACCAAA; *Cx45* reverse: CCCACCTCAAACACAGTCCT; for *Cx32* forward: AGGTGTGGCAGTGCCAGG; *Cx32* reverse: ACCACCAGCACCATGATTCTG; *ZO-1* forward: GTTTAGGAGCACCAAGTGC; *ZO-1* reverse: TCCTGTACACCTTTGCTGG; for *Occludin* forward: TGGCTGCTGCTGATGAATA; *Occludin* reverse: CATCCTCTTGATCTGCGATAAT. RT-PCR primer for *Clmp* to detect CLMP-encoding mRNA in ureteral tissue was: forward: ATCTCACCATGGCCTCCTC; reverse: GTGCTGAGTGTGGTTTCTGC. As control served: *Actb* forward: CGTGGGCCGCCCTAG; *Actb* reverse: CTTAGGGTTCAGGGGGGC.

### Biochemical methods

Determination of ions (sodium, potassium, calcium, chloride and magnesium), glucose, urea, creatinine or total protein in urine from adult wild type or *Clmp* mutants (mixed background) was performed by Labor28 (Berlin, Germany). SDS-PAGE and western blotting of urine was done according to standard procedures under non-reducing conditions with a goat anti-NGAL antibody (R&D Systems, #AF1857, 1:1000). To detect CLMP in brain tissue, membranes enriched by centrifugational forces were solubilized with 1% CHAPS in PBS supplemented with protease blockers (aprotinin, PMSF, leupeptin, pepstatin). Membrane proteins of the smooth muscle cell layer were solubilized in 1% SDS in Tris-buffered saline, pH 7.4, and protease blockers. Insoluble material was removed by centrifugation at 100,000 ***g***.

For cell surface labeling, the smooth muscle cell layer was peeled off from enteric tissue in PBS. Labeling was done with 2 mM Sulfo-NHS-LC-Biotin [sulfosuccinimidyl-6-(biotinamido) hexanoate; Thermo Scientific, #21335] in PBS at 4°C for 1 h followed by quenching with 100 mM glycin in PBS and several washing steps. Proteins were extracted in 1% SDS in Tris-buffered saline (pH 7.0) supplemented with protease blockers and non-solubilized components were removed by centrifugation at 100,000 ***g*** for 10 min. Biotinylated proteins were then enriched using streptavidin agarose resin (Thermo Scientific). Bound proteins were eluted by SDS PAGE sample buffer. The following antibodies were used for western blotting: rabbit anti-Connexin43 (Cell Signaling, #3512, 1:1000), rabbit anti-pan-Cadherin (Sigma, #C3678, 1:1000), rabbit anti-Connexin45 (Abcam, #AB16588, 1:1000), mAb anti-GAPDH (Novus Biologicals, #NB300-221, 1:7500), mAb anti-clathrin heavy chain (BD Transduction Laboratories, #610499, 0.1 µg/ml), mAb anti-caveolin (Transduction Laboratories, 0.1 µg/ml), rabbit 6504 (0.5 µg/ml; see below) and mAb anti-smooth muscle actin (Sigma, clone 1A4, #A5228, 0.1 µg/ml). Binding of antibodies or streptavidin-HRP (Sigma, 0.1 µg/ml) was determined by Clarity Western ECL solution (Bio-Rad) and Chemi-Doc imager (Bio-Rad). Image processing and analyses of western blots of Connexin43 were carried out using custom-written scripts for Fiji (http://rsbweb.nih.gov/ij/) or Quantity One (Bio-Rad). Mean gray values of bands of phosphorylated, non-phosphorylated and total Connexin43 were calculated from rectangular areas (Schindelin et al., 2012).

For the preparation of villi, intestinal segments were cut open and villi were scraped off by a brush in 5 mM EDTA/PBS and collected by centrifugation.

### Generation of antibodies to CLMP

Rabbit antibodies to murine CLMP were raised against a fusion protein composed of the extracellular region of mouse CLMP (aa residues 18-232) using vector pIg-plus and the heavy chain of human IgG1 (rabbit 101 and 102) or raised against the cytoplasmic segment obtained from a bacterial fusion protein containing GST and the cytoplasmic segment of mouse CLMP (aa residues 256-373) using vector pGEX-6P1 (GE Healthcare) (rabbit 6504). After removal of the GST portion, the cytoplasmic segment was further purified by ion exchange chromatography. The fusion proteins containing the extracellular domain of CLMP were generated in COS7 cells and purified by ProteinA affinity chromatography (GE Healthcare). Rabbits were injected five times with 50 µg each supplemented with Freund's adjuvant (Sigma) at fortnightly intervals. The IgG fractions were isolated by ProteinA affinity chromatography (GE Healthcare). The IgG fractions of rabbit 6504 or rabbit 102 were further affinity purified on columns containing the cytoplasmic segment of CLMP or the extracellular domain coupled to CNBr-activated Sepharose 4B (GE Healthcare), respectively.

### Immunohistology

Immunohistochemistry was performed on 4% paraformaldehyde-fixed 15 µm cryostat sections of tissue from the urinary and gastrointestinal tract using the following antibodies: mouse anti-neurofilament (Developmental Hybridoma Bank, #2H3, 1:200), mouse anti-smooth muscle actin (αSMA; Sigma, #A5228; 5 µg/ml), rat anti-cytokeratin 8 (Developmental Hybridoma Bank, #TROMA-I concentrate, 1:700), rabbit anti-Connexin43 (Cell Signaling, #3512, 1:200), rabbit anti-Connexin45 (Millipore, #AB1745, 1:100), sheep anti-Connexin45 (Abcam, #ab16588, 1:1000), rat anti-c-kit (Novus Biologicals, #NBP1-43359, mACK2, 1:1000), rabbit anti-ASAM (Bioorbyt, #orb100510), rabbit anti-CLMP (Sigma, #HPA002385, 1:100), mouse anti-Mucin2 (F-2, #sc-515032, 1 µg/ml), rabbit 102AP (0.5 µg/ml) and rabbit anti-ALDH1A2 (Abcam, #ab75674, 1:500). In addition to a standard staining procedure, a heat-induced antigen retrieval protocol was used to detect CLMP. Cell nuclei were stained by DAPI. Monoclonal antibodies and corresponding secondary antibodies were incubated on sections using the MOM kit (Vector Laboratories, BMK-2202).

Glycol methacrylate sections (5 µm) of urinary or gastrointestinal tract were performed for hematoxylin and eosin (HE) staining. All microscopic images were obtained at room temperature using an inverted LSM710 confocal microscope and ZEN acquisition software (Zeiss) or Biozero BZ-8100 microscope and BZ Analyzer software.

Analyses of connexin clusters detected by anti-connexin antibodies in confocal images of cryostat sections were done by Fiji software setting the threshold to RenyiEntropy routine; clusters were accepted between 0.05 and 1.00 µm^2^ (2-40 pixels) in the smooth muscle cell layer within the view field of 160.04×160.04 µm^2^.

### Electron microscopy

Pieces of duodenum from 4-week-old wild-type or CLMP-deficient mice were fixed in 4% formaldehyde/3% glutaraldehyde in 0.1 M cacodylate buffer overnight and post-fixed with 1% osmium tetroxide for 3 h according to Komuro (1989). After osmication, specimens were rinsed in distilled water and block stained with 5% uranyl acetate in distilled water for 3 h, dehydrated through a graded series of ethanol and embedded in Poly/Bed 812 (Polysciences, Inc., Eppelheim, Germany). Ultrathin sections were stained with uranyl acetate and lead citrate, and examined with an FEI Morgagni electron microscope and the iTEM software (EMSIS GmbH, Münster, Germany).

### Ureter explant cultures, time-lapse recordings of ureter, intrapelvic ink injections and analysis of gastrointestinal milk

Ureters from E15.5 embryos were cultured and imaged at the air-liquid interface on PET filter membranes (0.4 µm pore size, BD Falcon) containing DMEM/Ham's F12+GlutaMAX supplemented with 5 µg/ml transferrin, 100 µg/ml penicillin and 100 U/ml streptomycin at 37^°^C in an atmosphere of 5% CO_2_ as detailed by Cain et al. (2011). Time-lapse recordings were done at days *in vitro* (DIV) 5 using an inverted LSM 710 confocal microscope (Zeiss) equipped with temperature and CO_2_ controller. Time-lapse images of ureters were acquired every 270 ms for a total duration of 20 min. Peristalsis was then quantified by measuring the lumen diameter at fixed points throughout the contraction period.

Intrapelvic ink (12.5% in H_2_O; Pelikan) injections were done in E18.5 embryos with pulled glass pipettes using a micromanipulator and a Cell Tram oil injector (Eppendorf) (Cain et al., 2011). The analysis of transport of milk in the gastrointestinal tract of P0.5 mice was done from photographic images processed in ImageJ (NIH).

### Image acquisition of the contraction activity of the duodenum, jejunum or colon and calculation of spatiotemporal maps

The duodenum, jejunum or colon from wild-type, heterozygotes or *Clmp*^−/−^ mice at P40-P44 killed by cervical dislocation was rapidly removed and placed in an organ bath at 37°C superfused with Krebs solution and bubbled with 95% O_2_-5% CO_2_ (Swaminathan et al., 2016). The segments were secured at both ends. The motility patterns of single points of isolated segments of the duodenum and jejunum in the longitudinal and transversal axes were quantified at specified positions via a method developed similarly to that in Lentle et al. (2007). Images of 540×540 pixels were captured subsequently from an on-screen recording of live mode microscopy (Zeiss Stemi SV11, Camera Zeiss AxioCam HRc) at a rate of 25 frames/second. An algorithm written in MATLAB was applied to consecutive frames employing normalized cross correlation to track the displacement of a 35×35 pixel square (0.73×0.73 mm) enclosing a unique pattern of vascular arcade on the serosal surface. The coordinates of the square's position in each frame was attained given the maximum value of normalized cross correlation coefficient (roughly 1) and saved in an array for further analysis. The obtained coordinates were plotted individually in either the horizontal or vertical axis over time, giving the motility pattern of the intestinal segment at that specific point of interest in the horizontal and vertical planes. The average amplitude and frequency of the movements were estimated consequently from these motility graphs (Lentle et al., 2007).

For spatiotemporal maps, movements of intestinal segments were recorded as video files in mp4 format using a video camera (SJCAM SJ5000, 30 frames/s, 1920×1080 pixels). The image processing, analysis, visualization and algorithm development for generation of spatiotemporal maps were all proceeded in MATLAB (R2016a) in accordance with the approach of Benard et al. (1997).

### Isometric force studies

Fundus, LES and PS were transferred to Krebs-Henseleit solution bubbled with 95% O_2_ and 5% CO_2_. Fundus strips were mounted longitudinally on fixed segment support pins in two four-chamber myographs (Myograph 610; Danish Myo Technology, Aarhus, Denmark) containing 5 ml of the Krebs-Henseleit solution. Resting tension was set to 3 mN. Strips were precontracted with CCh (0.1 µmol/l). Relaxation was induced with DEA-NO as indicated. IBMX (100 µmol/l) was added at the end of each experiment to determine maximal relaxation. The LES was fixed as rings on segment support pins to measure circular muscle tension. Resting tension was set to 2 mN. The strips were contracted with CCh (1 µmol/l) and, subsequently, DEA-NO was applied as indicated. Rings of the PS were fixed on segment support pins to measure phasic contractions of circular muscle. Maximal contractile force of each sphincter was determined from recordings over 30 min (Groneberg et al., 2015, 2011).

### EFS and calcium imaging

Fundus strips as well as rings from duodenum and colon were mounted and the resting tension was set to 1 mN. EFS (0.5 ms, 1-8 Hz, 10 s, supramaximal voltage) was applied through two platinum wire electrodes (5 mm distance).

Changes in intracellular Ca^2+^-concentrations were recorded in cultured ureter loaded with the Ca^2+^-indicator fura-2 for 1 h as described (Unsoeld et al., 2008). Duodenum was cut open, the villi-containing side was put facing downwards and, for calcium imaging, strips were secured at several points with tungsten needles.

### Statistics

Numerical data are presented as means±s.e.m. or as means±s.d. (qRT-PCR). Data were analyzed by SigmaStat (V11.0, Systat Software, Inc.). Data were first analyzed by the Kolmogorov–Smirnov test whether they are normally distributed followed by analysis of significance using Mann–Whitney *U*-test or by a *t*-test. Isometric force studies were analyzed by the Mann–Whitney *U*-test using GraphPad Prism software version 4.00 (GraphPad Software, San Diego, CA, USA). The survival rates of CLMP-deficient mice on the mixed and C57BL/6 genetic background were compared using the log-rank test, Breslow test and Tarone–Ware test. Significance levels were indicated as *P≤0.05, **P≤0.01 or ***P≤0.001.

## Supplementary Material

Supplementary information
