# Supplementary material for: The IgCAM CLMP regulates expression of Connexin43 and Connexin45 in intestinal and ureteral smooth muscle contraction in mice
Source: Dis Model Mech. 2018 Feb 1;11(2):dmm032128. doi: 10.1242/dmm.032128 (PMC5894946; doi:10.1242/dmm.032128)
Supplement: Supplementary information [file dmm-11-032128-s1.pdf]

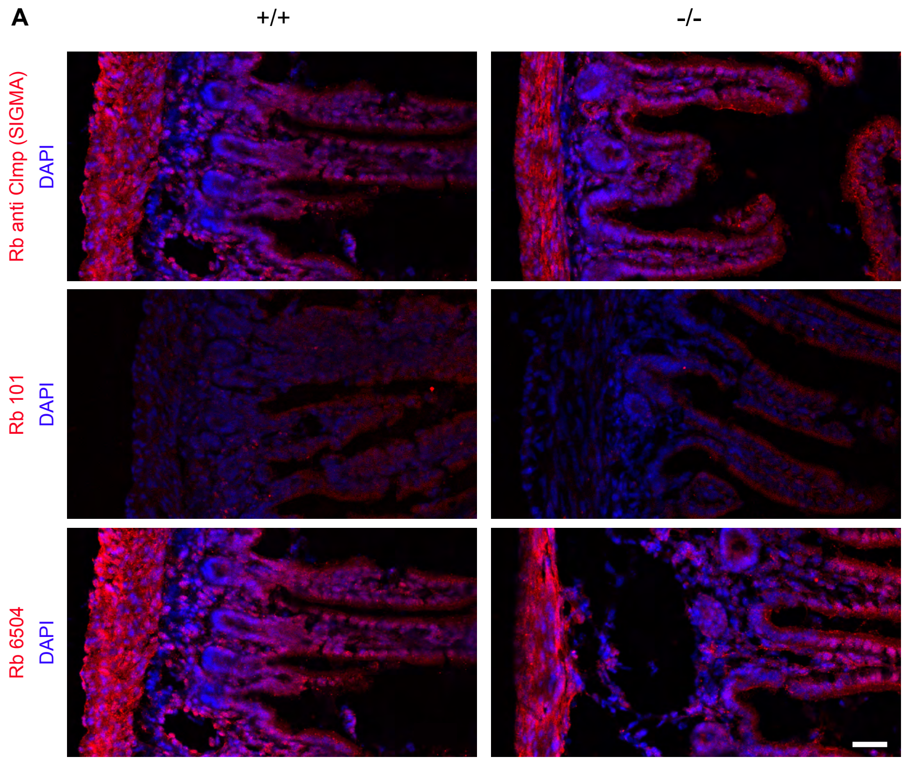

B

```
mouse    1 rkskdryeeedrpneiredaeaprarlvkpssssgsrssrsgssstrst  50
           ||.|:|||||:|||||||||||||:|||||||||||||||||||||||||
human    1 rkdkeryeeeerpneiredaeapkarlvkpssssgsrssrsgssstrst  50

mouse   51 gnsasrsqrtlsseaapqqpglapqaysligpevrgsepkkvhhttltka  100
           .|||||||||||::||| |||||.|||||:||||||||||||||..||||
human   51 ansasrsqrtlstdaap-qpglatqayslvgpevrgsepkkvhhanltka  99

mouse  101 ettlsttpsqskafqtv    117
           |||.|.
human  100 ettpsm-----------    105
```

Figure S1

Several antibodies to CLMP are improper in immunohistology to detect CLMP in the intestine.

A) No specific staining was obtained in wild type tissue if compared with knockout intestine using commercially available antibodies [rabbit anti-ASAM (Bioorbyt #orb100510) or rabbit anti-CLMP (Sigma #HPA002385, 1:100). Only the latter is shown here.], rabbit antibody (#6504) directed to the cytoplasmic segment or rabbit antibody 101 against the extracellular region of CLMP. The tissue from 4 weeks old animals was fixed in PFA for 1 hour. In addition an antigen retrieval protocol was tested. Scale bar, 20 µm.

B) Alignment of the amino acid sequence of the cytoplasmic segments of human and mouse CLMP indicating high identity and predicting cross-reactivity of anti-hCLMP antibodies to detect murine CLMP.

Disease Models & Mechanisms • Supplementary information

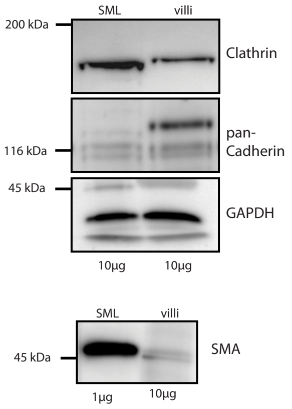

Figure S2

Enrichment of smooth muscle cell layer and villi from intestine.

Western blotting demonstrating the enrichment of the smooth muscle cell layer or villi preparations from intestine using antibodies to pan-Cadherin (a marker for the villi) and an antibody to smooth muscle actin. In the upper panel 10 µg of protein per lane was loaded. GAPDH and clathrin served as additional loading control. In the lower panel 1 µg of protein was loaded from the smooth muscle layer and 10 µg from villi. Molecular mass markers are indicated at the left of the panels.

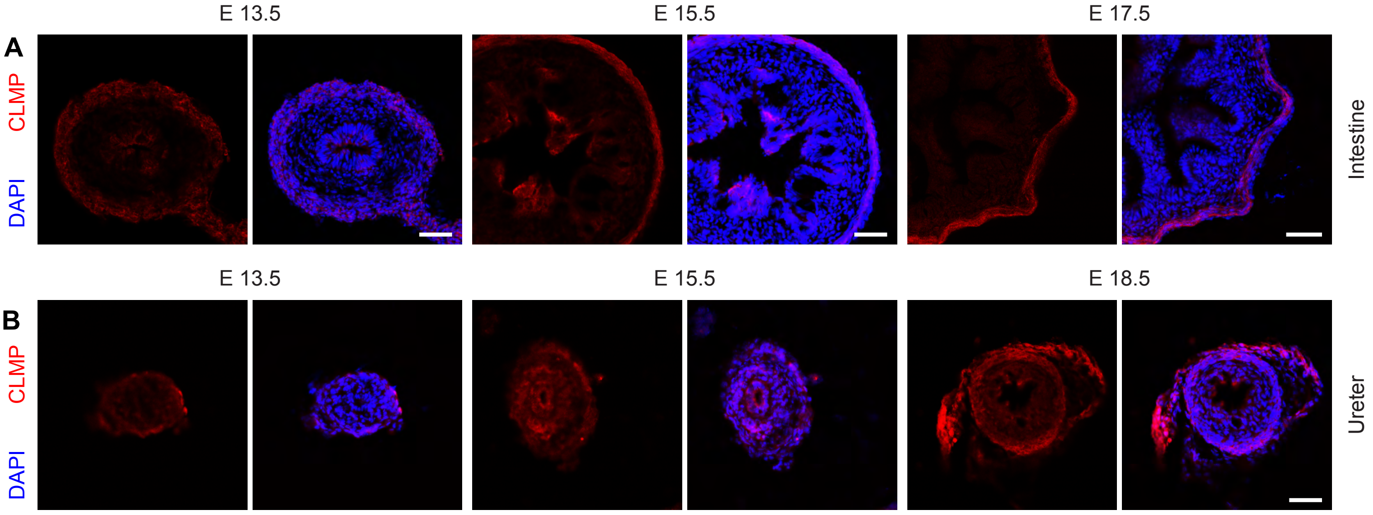

Figure S3

Localization of CLMP in the embryonic intestine and ureter.

Localization of CLMP in cross sections of intestine and ureter at different embryonic stages using affinity purified antibody 102 to the extracellular domain of mCLMP. CLMP is primarily localized in the developing smooth muscle layer and more weakly in the mucosa of the intestine and in the developing mesentery (see left panel of the intestine row). In ureter sections CLMP is also primarily expressed in the developing smooth muscle layer and in the surrounding fat tissue. Sections were counter-stained with DAPI. Bar, 50 µm.

Disease Models & Mechanisms • Supplementary information

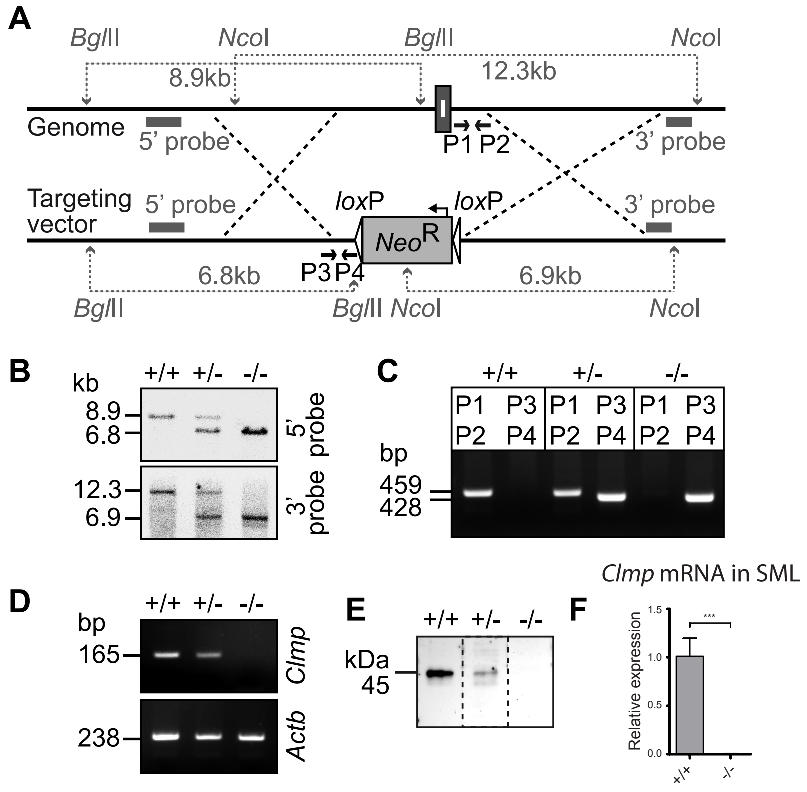

Figure S4

Generation of CLMP-deficient mutant mice.

A) Targeting strategy for the generation of CLMP-deficient mice. The neomycin cassette, *lox*P sites and the *Bgl*II and *Nco*I restriction sites are depicted. Black bars represent the sequences used as 5' or 3' probes for Southern blot analysis. Arrows P1, P2, P3 and P4 show the positions of the primers used for genotyping by PCR. B) Southern blot after digestion of genomic DNA with *Bgl*II or with *Nco*I using 5' or 3' probes, respectively. C) PCR analysis of genomic DNA with primers P1 and P2 or P3 and P4 for amplification of the wild type or the mutant allele, respectively. A 459 bp product is generated from wild type allele and a 428 bp product from the targeted allele. D) RT-PCR of RNA extracted from brain tissue reveal the absence of CLMP encoding mRNA in CLMP- deficient mice. *Actb* amplification was used as control to verify integrity of total RNA isolation and cDNA reverse transcription. E) Western blot of membrane enriched fractions from brain tissue using an antibody to the cytoplasmic domain of CLMP demonstrate the absence of CLMP in mutant mice. A 46 kDa band is revealed in wild type or heterozygous mice. F) Quantitative RT-PCR of intestinal smooth muscle tissue (4 weeks old) from wild type and CLMP knockout mice. A 1050 fold higher value was detected in wild type in comparison to knockout indicating the absence of mRNA encoding *Clmp* in the CLMP knockout (p=0.0007).

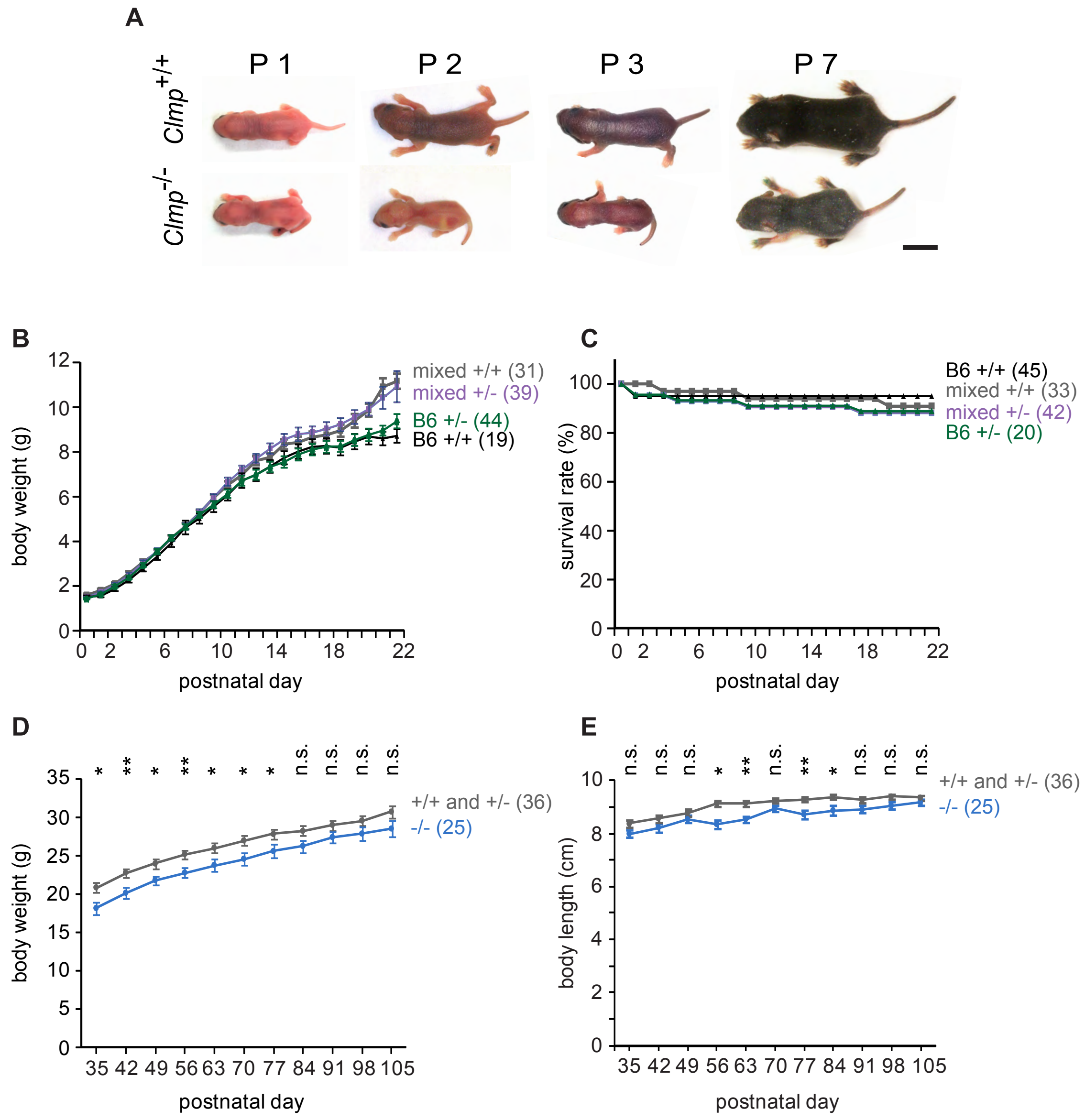

Figure S5

Appearance of CLMP-deficient mice at early postnatal stages.

A) Photographs of examples of CLMP-deficient and control littermates at early postnatal stages are shown. Scale bar, 1 cm.

B and C) Wild type animals (black, B6 background and gray, mixed background) do not differ from heterozygous littermates (green, B6 background and violet, mixed background) in their development of body weight (B) and survival rate (C). All comparisons between wild type and heterozygous genotypes within each strain are not significant. Numbers in brackets indicate numbers of analyzed animals.

D and E) Knockout mice of the mixed background strain (blue) at post-weaning stages display a significant delay in gain of body weight as compared to control littermates (pooled data of wild type and heterozygous mice; gray), whereas naso-anal body length (D) is almost comparable between control and knockout.

Disease Models & Mechanisms • Supplementary information

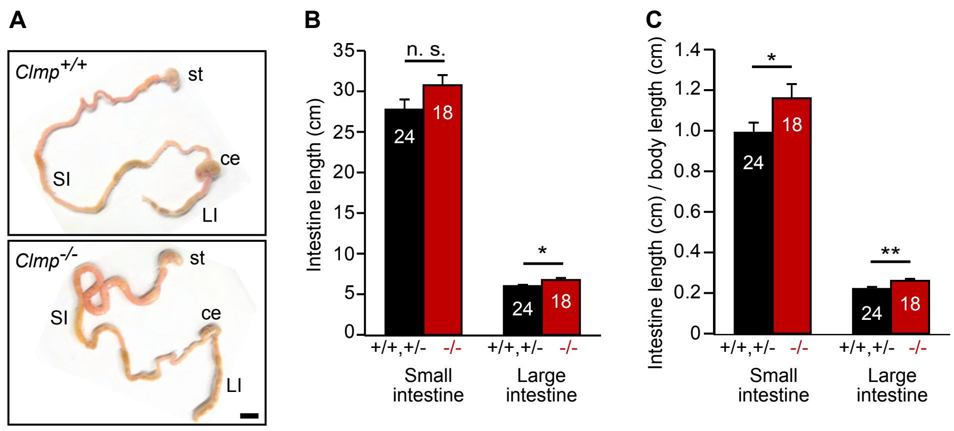

Figure S6

Intestinal length from adult CLMP-deficient mice does not differ from wild types.

Intestines of adult mice (mixed genetic background) were dissected; gut lengths were measured and normalized to body weights. A) Examples, B) total intestine length and C) total intestine length related to body length. Numbers in brackets indicate numbers of analyzed animals. SI, small intestine; LI, large intestine; st, stomach; ce, cecum. Scale bar, 1 cm.

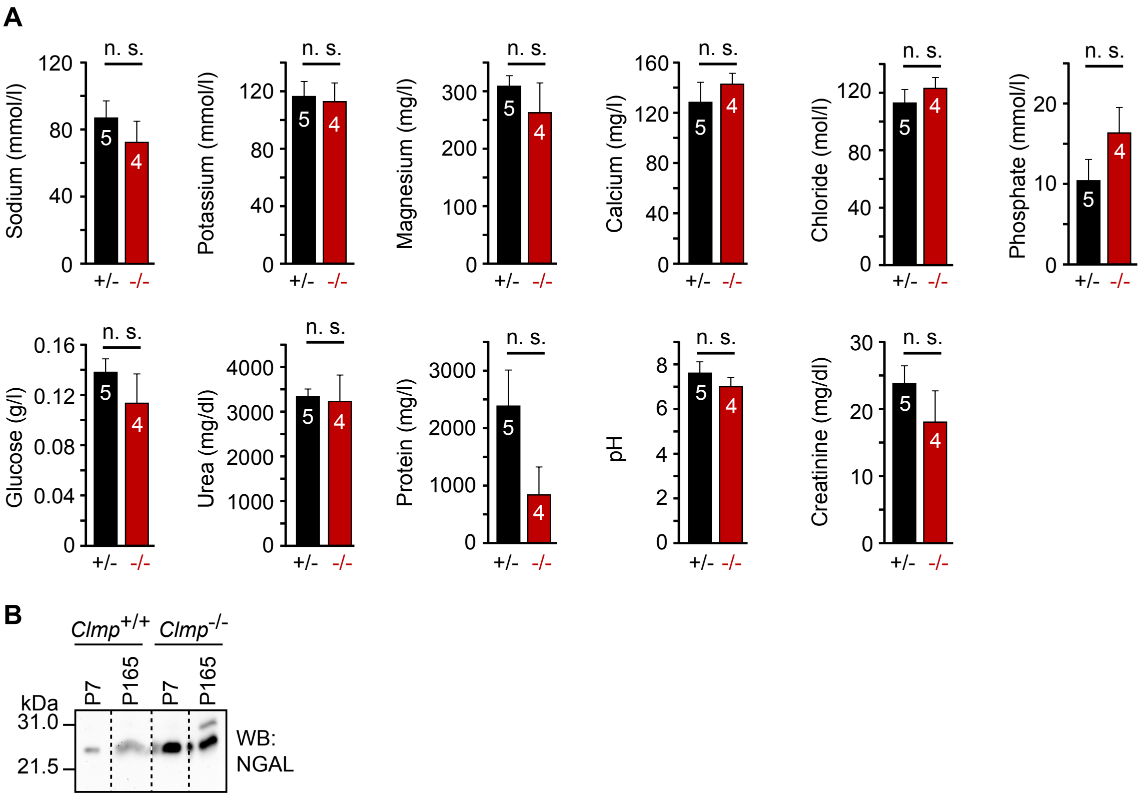

Figure S7

Urine composition and increased NGAL in CLMP-deficient mice.

A) Urine analysis of adult *Clmp* mice (mixed genetic background) did not show any significant alterations in the levels of electrolytes, glucose, urea, protein, pH and creatinine in the absence of CLMP. Numbers in brackets indicate numbers of analyzed animals.

B) The concentration of urinary NGAL (neutrophil gelatinase-associated lipocalin) which is rapidly and massively induced during renal injury was increased in *Clmp* mutant mice (mixed background). Urine was collected by bladder puncture at P7 or P165. Equal volumes were loaded on a non-reducing SDS-PAGE and analysed by Western blotting.

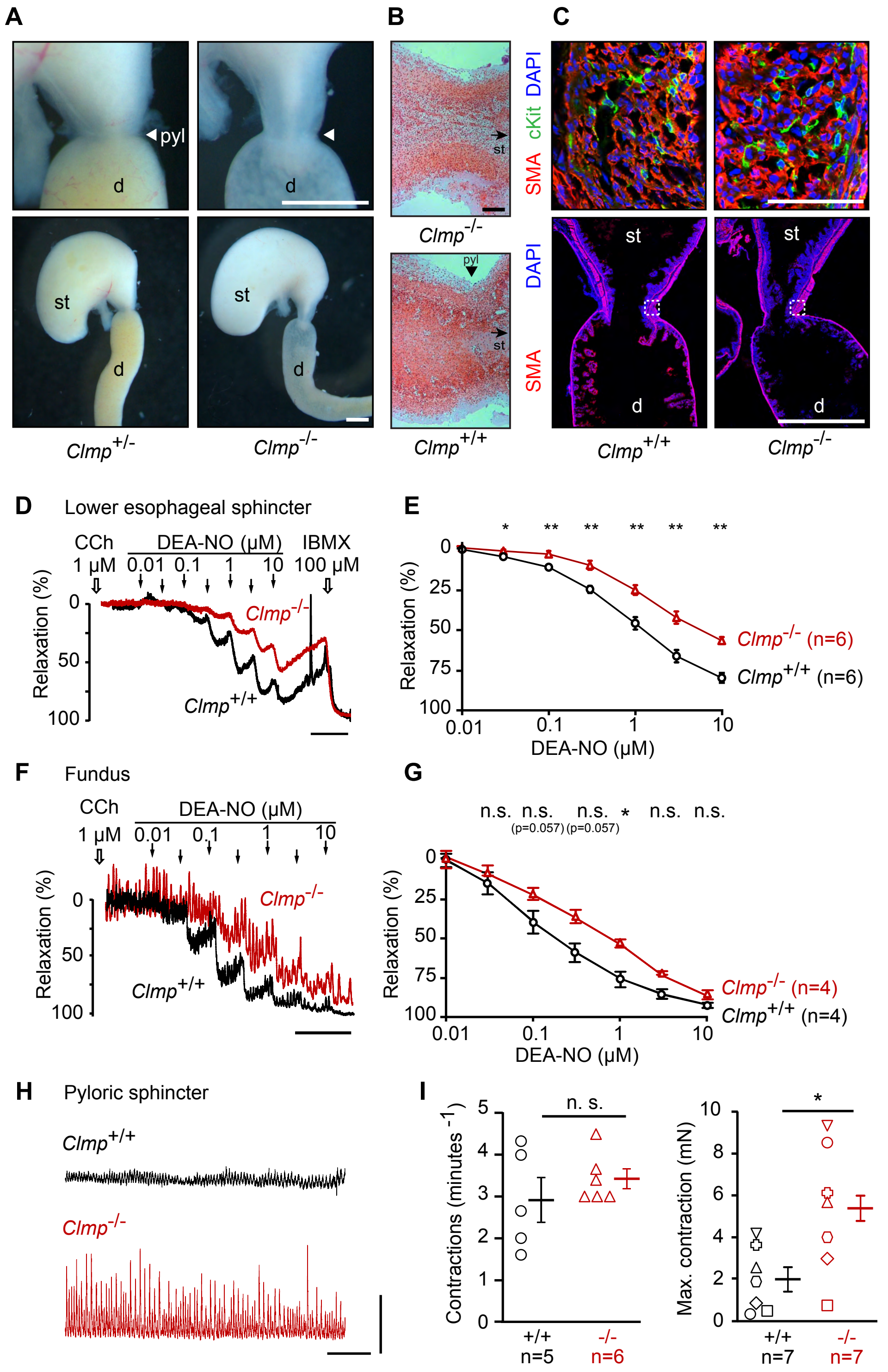

Figure S8

Increased muscle tone but no physical barriers in the CLMP-deficient gastrointestinal tract.

A) Gastrointestinal dissections from P0.5 pups showing stomach (st) and duodenum (d) with pyloric region (pyl; arrowhead). A different shape at the transition zone from the antrum to the duodenum is observed. Seven out of eight animals showed this elongated shape. Scale bars, 1 mm. B) A narrowing of the pyloric opening was not detected in longitudinal HE-stained sections from P0.5 mice excluding a pyloric stenosis (arrow head). The arrow points to the direction of the stomach. Scale bar, 100 µm. C) Sections of the P0.5 pylorus reveal a normal composition and thickness of cell layers including the presence of c-Kit-positive and smooth muscle cells. The small square in the lower images represents higher magnifications shown in the upper panels. Lower panels scale bar, 1 mm; upper panels scale bar 100 µm.

D and E) Representative traces for NO-induced lower esophageal sphincter (LES) relaxation. LES from Clmp+/+ and Clmp-/- mice were pre-contracted with carbachol (CCh, 1 µmol/L) and subsequently relaxed with increasing concentrations of DEA-NO. IBMX was applied to determine maximal relaxation. Scale bar, 5 min. (E) Statistical analysis of LES data. F) and G) Fundus strips from Clmp+/+ and Clmp-/- mice were pre-contracted with CCh (1 µmol/L) and subsequently relaxed with increasing concentrations of DEA-NO. Data shown are mean ± SEM of n = 4 per genotype.

H) Spontaneous rhythmic contractions of pyloric sphincter from P48 days old CLMP+/+ and CLMP-/- mice. Horizontal scale bar, 5 min; vertical scale bar 2 mN.

I) Analysis of contractions per minute and analysis of maximal single contractions from P45-P50 old mice. Identical symbols indicate Clmp+/+ and Clmp-/- mice siblings measured on the same day (p=0.0379; Mann-Whitney-U-Test).

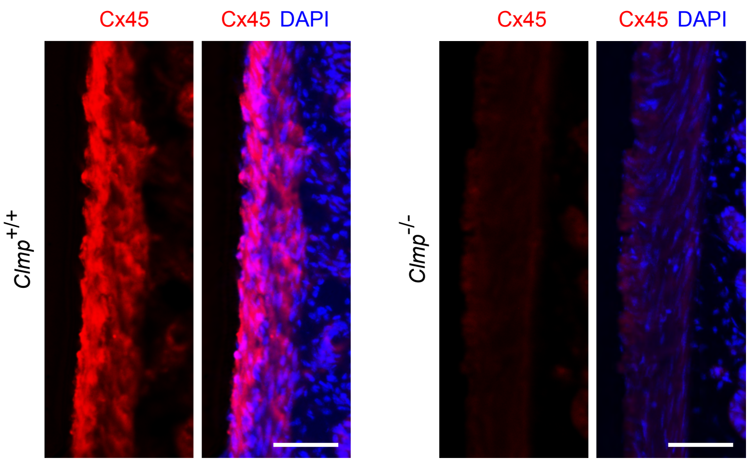

Figure S9

Localization of Connexin45 in the duodenum in in wild type and CLMP-deficient mice.

A and B) Connexin45 plaques are reduced in the circular smooth muscle cell layer of CLMP-deficient duodenum (A). Quantitative data are presented in Figure 7D. Bar, 50 µm.

		Clmp$^{+/+}$	*Clmp*$^{+/-}$	*Clmp*$^{-/-}$
mixed background	number of pups	63	115	40
	percentage	28.90 %	52.75 %	18.35 %
	Chi²	1.3257	0.3303	3.8578
B6 background	number of pups	71	136	49
	percentage	27.73 %	53.13 %	19.14 %
	Chi²	0.7656	0.5000	3.5156

Table S1

Configural frequency analysis of recovery of Mendelian ratio in newborn pups (P0.5) from heterozygous-to-heterozygous mating. Animal numbers with a Chi² value > 3.841 are under-represented. Monitoring was done at noon.

Organ	Genotype	Organ weight (g)		Ratio organ weight/ body weight		Ratio organ weight/ body length	
**Brain**	Ctrl. (n = 15)	0.601 ± 0.015	P = 0.005	0.022 ± 0.001	P = 0.586	0.066 ± 0.008	P = 0.122
	KO (n = 10)	0.531 ± 0.016	** [*t*]	0.021 ± 0.001	n.s. [*t*]	0.061 ± 0.005	n.s. [*t*]
**St. + Int.**	Ctrl. (n = 18)	3.322 ± 0.150	P < 0.001	0.124 ± 0.005	P < 0.001	0.365 ± 0.014	P < 0.001
	KO (n = 12)	**4.563 ± 0.210**	*** [*t*]	**0.183 ± 0.008**	*** [*t*]	**0.529 ± 0.022**	*** [*t*]
**Heart**	Ctrl. (n = 16)	1.181 ± 0.008	P = 0.056	0.007 ± 0.000	P = 0.353	0.020 ± 0.001	P = 0.184
	KO (n = 11)	0.155 ± 0.010	n.s. [MW]	0.006 ± 0.000	n.s. [*t*]	0.018 ± 0.001	n.s. [*t*]
**Kidney**	Ctrl. (n = 36)	0.224 ± 0.007	P < 0.001	0.008 ± 0.000	P < 0.001	0.025 ± 0.001	P < 0.001
	KO (n = 22)	**0.837 ± 0.062**	*** [MW]	**0.033 ± 0.002**	*** [MW]	**0.097 ± 0.007**	*** [MW]
**Liver**	Ctrl. (n = 16)	1.389 ± 0.058	P = 0.217	0.050 ± 0.002	P = 0.694	0.152 ± 0.006	P = 0.838
	KO (n = 11)	1.292 ± 0.077	n.s. [MW]	0.051 ± 0.003	n.s. [*t*]	0.150 ± 0.009	n.s. [*t*]
**Lung**	Ctrl. (n = 17)	0.245 ± 0.008	P = 0.003	0.009 ± 0.000	P = 0.422	0.026 ± 0.001	P = 0.056
	KO (n = 11)	0.210 ± 0.006	** [*t*]	0.008 ± 0.000	n.s. [*t*]	0.024 ± 0.001	n.s. [*t*]
**Spleen**	Ctrl. (n = 16)	0.114 ± 0.010	P = 0.049	0.004 ± 0.000	P = 0.312	0.013 ± 0.001	P = 0.079
	KO (n = 11)	0.087 ± 0.010	* [MW]	0.003 ± 0.000	n.s. [MW]	0.010 ± 0.001	n.s. [MW]

Table S2

Wet weight analysis of organs of the *Clmp*-deficient mice (mixed background) at P97 - P140.

St., stomach; Int., intestine; Ctrl., control; KO, knockout; *t*, *t*-test; MW, Mann-Whitney-*U* test; n.s., not significant; * indicating P ≤ 0.05, ** indicating P ≤ 0.01 and *** indicating P ≤ 0.001. Significant changes in kidney and the gastrointestinal tract are printed in bold.

Disease Models & Mechanisms • Supplementary information

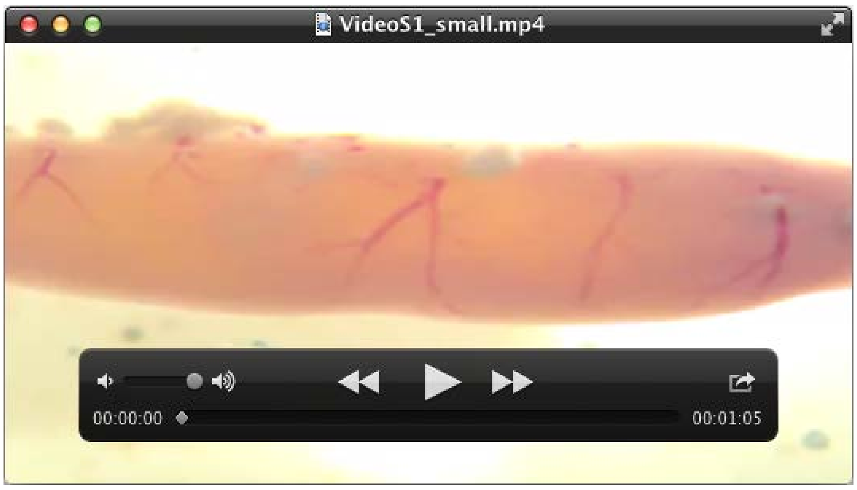

Video S1

Movement of a duodenal segment in an organ bath from wild type, real time.

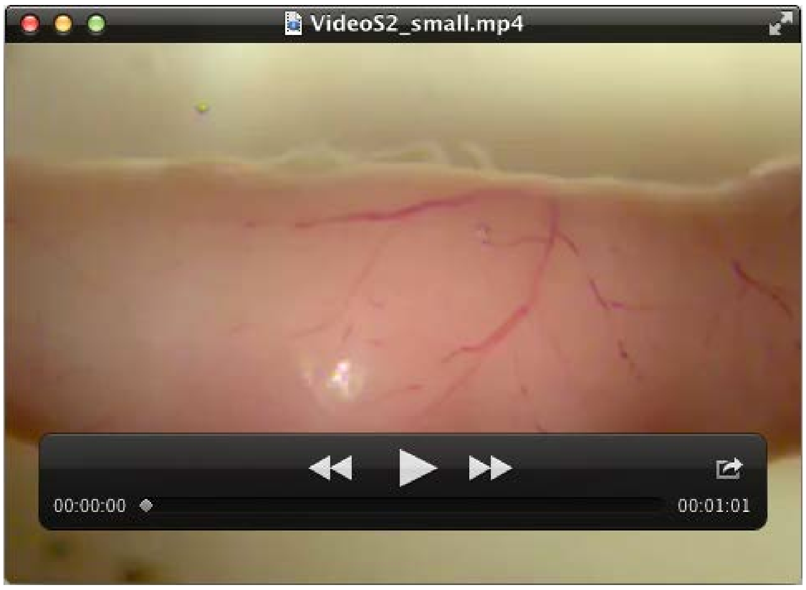

Video S2

Movement of a duodenal segment in an organ bath from CLMP knockout, real time.

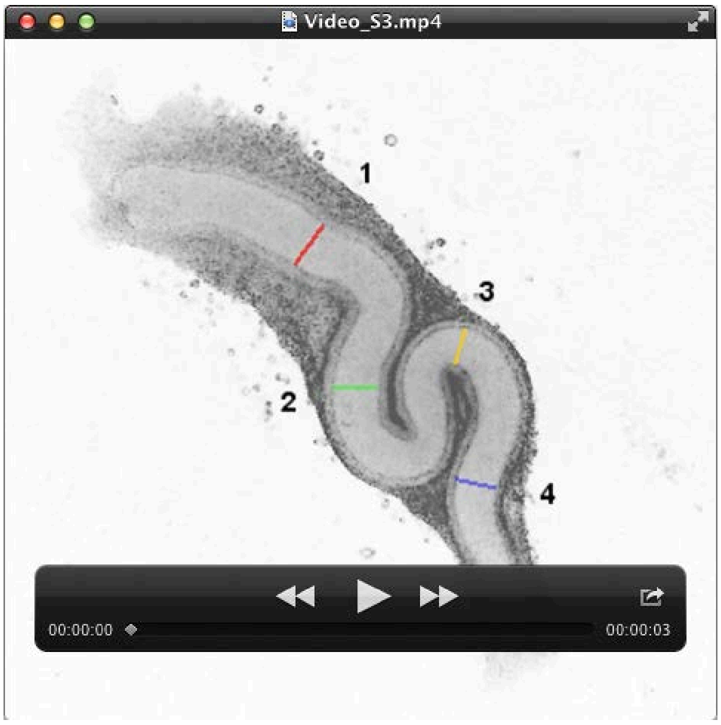

Video S3

Contraction of a cultured ureter from wild type, time lapse at 270 ms intervals.

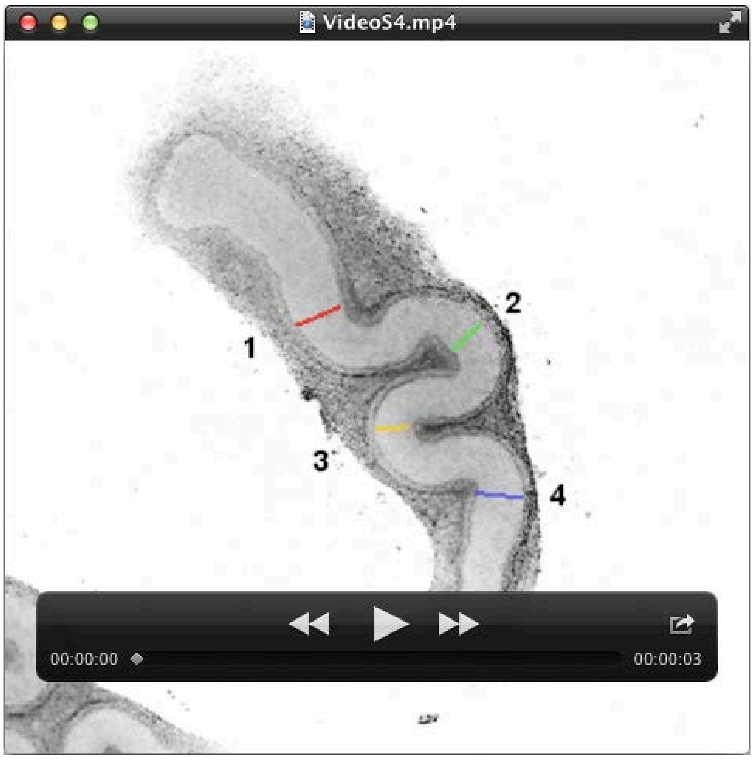

Video S4

Contraction of a cultured ureter from CLMP knockout, time lapse at 270 ms intervals.
